# Comparative analysis of genome-scale, base-resolution DNA methylation profiles across 580 animal species

**DOI:** 10.1038/s41467-022-34828-y

**Published:** 2023-01-16

**Authors:** Johanna Klughammer, Daria Romanovskaia, Amelie Nemc, Annika Posautz, Charlotte A. Seid, Linda C. Schuster, Melissa C. Keinath, Juan Sebastian Lugo Ramos, Lindsay Kosack, Ann Evankow, Dieter Printz, Stefanie Kirchberger, Bekir Ergüner, Paul Datlinger, Nikolaus Fortelny, Christian Schmidl, Matthias Farlik, Kaja Skjærven, Andreas Bergthaler, Miriam Liedvogel, Denise Thaller, Pamela A. Burger, Marcela Hermann, Martin Distel, Daniel L. Distel, Anna Kübber-Heiss, Christoph Bock

**Affiliations:** 1 CeMM Research Center for Molecular Medicine of the Austrian Academy of Sciences, Vienna, Austria; 2 Gene Center and Department of Biochemistry, Ludwig-Maximilians-Universität München, Munich, Germany; 3 Research Institute of Wildlife Ecology, University of Veterinary Medicine Vienna, Vienna, Austria; 4 Ocean Genome Legacy Center, Northeastern University Marine Science Center, Nahant, USA; 5 Department of Biology, University of Kentucky, Lexington, KY USA; 6 Max Planck Research Group Behavioral Genomics, Max Planck Institute for Evolutionary Biology, Plön, Germany; 7 Children’s Cancer Research Institute, St. Anna Kinderkrebsforschung, Vienna, Austria; 8 Institute of Marine Research (IMR), Bergen, Norway; 9 Medical University of Vienna, Center for Pathophysiology Infectiology and Immunology, Institute of Hygiene and Applied Immunology, Vienna, Austria; 10 Institute of Avian Research, An der Vogelwarte, Wilhelmshaven, Germany; 11 Department for Pathobiology, University of Veterinary Medicine Vienna, Vienna, Austria; 12 Medical University of Vienna, Department of Medical Biochemistry, Vienna, Austria; 13 Medical University of Vienna, Institute of Artificial Intelligence, Center for Medical Data Science, Vienna, Austria

**Keywords:** Epigenomics, Gene regulatory networks, Genome evolution

## Abstract

Methylation of cytosines is a prototypic epigenetic modification of the DNA. It has been implicated in various regulatory mechanisms across the animal kingdom and particularly in vertebrates. We mapped DNA methylation in 580 animal species (535 vertebrates, 45 invertebrates), resulting in 2443 genome-scale DNA methylation profiles of multiple organs. Bioinformatic analysis of this large dataset quantified the association of DNA methylation with the underlying genomic DNA sequence throughout vertebrate evolution. We observed a broadly conserved link with two major transitions—once in the first vertebrates and again with the emergence of reptiles. Cross-species comparisons focusing on individual organs supported a deeply conserved association of DNA methylation with tissue type, and cross-mapping analysis of DNA methylation at gene promoters revealed evolutionary changes for orthologous genes. In summary, this study establishes a large resource of vertebrate and invertebrate DNA methylomes, it showcases the power of reference-free epigenome analysis in species for which no reference genomes are available, and it contributes an epigenetic perspective to the study of vertebrate evolution.

## Abstract (web summary)

DNA methylation is involved in regulatory processes throughout the animal kingdom. Here, the authors map DNA methylation in 535 vertebrates and 45 invertebrates, establishing a reference dataset for cross-species analysis and exploring epigenetic variation across vertebrate evolution.

## Introduction

DNA methylation at the fifth carbon position of cytosines (5-methyl-cytosine) provides an epigenetic layer of genome regulation that does not involve changes in the DNA sequence. In vertebrates, DNA methylation occurs preferentially at palindromic CpG dinucleotides, where it marks both strands symmetrically. It is essential for genome integrity and contributes to the silencing of transposable elements^1^. Moreover, it is involved in the regulation of many biological processes associated with multicellular life^2^, including development^3^, cell differentiation^4^, and maintenance of cellular identity^5,6^. DNA methylation has been studied extensively in the context of diseases such as cancer^7,8^, metabolic diseases^9^, autoimmune disorders^10,11^, and for its role in aging^12^. From an evolutionary perspective, DNA methylation and its associated enzymes (most notably the DNA methyltransferases that “write” DNA methylation) are present throughout the animal kingdom, although they have been lost in certain species including the model organism *Caenorhabditis elegans*^13^. DNA methylation has also been implicated in speciation^14^ and in the response to environmental influences^15,16^. The study of DNA methylation in a broad range of species is expected to contribute to our understanding of evolution.

Genome-wide DNA methylation patterns vary widely across species. Pioneering research in the early 1980s compared global levels of DNA methylation across several animal species^17,18^, which revealed major differences between vertebrates and invertebrates^19^. Moreover, considerable variability was observed among vertebrates^20–23^. While these initial studies relied on methylation-specific restriction enzymes or on chromatography-based methods, more recent investigations used next-generation sequencing to determine DNA methylation patterns in 17 eukaryotic species (which included two vertebrates)^24^, in 13 animal species (five invertebrate and seven vertebrate species)^25^, in seven vertebrate species^26^, and in eight mammalian species^27^. High-resolution DNA methylation maps enabled initial analyses of the evolutionary relationship between DNA methylation and the underlying DNA sequence^28–30^. These previous studies were however limited to a small number of species, while an ideal study would cover many species across all branches of vertebrate evolution—such that each species becomes a complex data point in a truly integrative analysis of DNA methylation in its evolutionary context.

In humans, where DNA methylation has been studied in most detail, a strong correlation exists between the patterns of genomic DNA sequence and local DNA methylation^31,32^. CpG-rich genomic regions (including many promoters and enhancers) tend to be unmethylated, except where they overlap with evolutionarily recent transposable elements^33^ or are subject to mechanisms of regulatory repression that involve DNA methylation^34^. In contrast, CpG-poor genomic regions tend to be highly methylated, except where they overlap with active transcription factor binding sites^35^ or in areas of large-scale DNA methylation erosion as observed in cancer and ageing^36,37^. The genome-wide correlation between DNA methylation and DNA sequence has enabled the prediction of locus-specific DNA methylation levels based on the underlying DNA sequence, focusing on CpG islands and gene promoters^38,39^ and on individual CpG dinucleotides^40,41^. Consistent with this genetic basis of DNA methylation, differences in the genomic DNA sequence between individuals have been linked to differences in DNA methylation^42,43^. Nevertheless, human primary samples tend to cluster according to tissue type rather than according to the sample donor^44^, indicating that DNA methylation differences between human individuals are generally less pronounced than tissue-specific differences.

To investigate DNA methylation beyond the human genome and in the broad context of vertebrate evolution, we established genome-scale DNA methylation profiles at single-base resolution across a wide range of vertebrate and invertebrate species, covering all vertebrate classes and several proximal invertebrate classes. Primary tissue or DNA samples were obtained from biobanks and other sources comprising mainly wildlife and zoo animals. We included heart and liver samples wherever possible, to allow for tissue-matched comparisons across species. In addition, other tissues such as lung, gills, fin, spleen, brain, lymph node, muscle, kidney, and skin were included in a species-specific manner. Samples were selected to prioritize healthy adults and to balance the male-to-female ratio, aiming for two to four individuals per species. This sampling strategy allowed us to cover a large number of species, consistent with our study’s focus on analyzing trends that hold across multiple species, rather than on the in-depth investigation of DNA methylation regulation in individual species.

DNA methylation profiling was performed using an optimized version of the reduced representation bisulfite sequencing (RRBS) assay^45–47^. Our assay enriches for CpG-rich regulatory regions but also covers many other parts of the genome, including exons, introns, intergenic regions, and repetitive elements; and it measures DNA methylation both at CpG sites and non-CpG sites in the genome. We analyzed our RRBS dataset using reference-genome independent bioinformatic methods^45^, allowing us to include many species that do not currently have a published reference genome and to avoid biases due to the different quality of available reference genomes. We previously validated this approach in a head-to-head comparison of reference-free and reference-based analysis in three species^45^. Moreover, as part of this study we confirmed that RRBS is indeed suitable for cross-species analysis, using in silico simulations of RRBS coverage based on existing reference genomes, comparison to whole genome bisulfite sequencing (WGBS) data, and reference-based analysis in a subset of samples.

Our full dataset comprises 2443 DNA methylation profiles covering 580 animal species (535 vertebrates and 45 invertebrates). Based on this dataset, we identified a quantitative, predictive association of DNA methylation and the underlying genomic DNA sequence that was shared between vertebrate and invertebrate species. We observed two major transitions along the evolutionary axis: one between vertebrates and invertebrates and one between amphibians and reptiles. We also investigated tissue-specific and inter-individual differences in DNA methylation. For fish, birds, and mammals, tissue-specific differences were more pronounced than inter-individual differences, but for invertebrates, reptiles, and amphibians, both factors explained a similar share of the observed variance in DNA methylation. By analyzing transcription factor binding sites in differentially methylated regions between heart and liver tissue throughout vertebrate evolution, we identified a deeply conserved association of DNA methylation with tissue identity. Finally, cross-mapping to existing reference genomes identified characteristic evolutionary trends in DNA methylation at gene promoters.

In summary, this study contributes an epigenetic perspective to the investigation of vertebrate evolution, and establishes a major resource for dissecting the role of DNA methylation in vertebrates and invertebrates. Moreover, our results emphasize the feasibility and value of including epigenome profiling in ongoing initiatives to map all vertebrate genomes^48^, and provide a starting point for untangling how the complex interplay of DNA sequence patterns and DNA methylation has contributed to the evolution of vertebrate genomes.

## Results

### An atlas of DNA methylation across 580 animal species

**Fig. 1:**
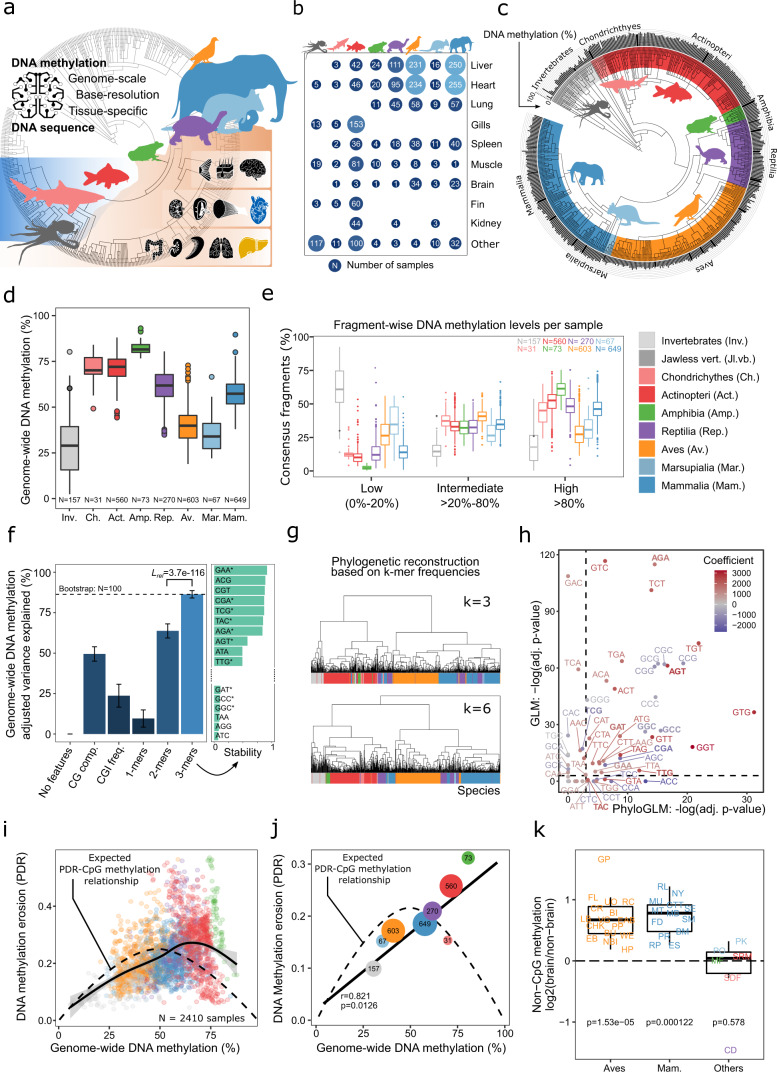
An atlas of DNA methylation comprising 580 animal species reveals global links between genomes and epigenomes throughout vertebrate evolution. **a** Visual summary of the study. The cross-species atlas comprises 2443 genome-scale DNA methylation profiles covering 580 animal species (535 vertebrates and 45 invertebrates). The animal silhouettes show one species per taxonomic group: Octopus (invertebrates), shark (cartilaginous fish), carp (bony fish), frog (amphibians), tortoise (reptilians), pigeon (birds), wallaby (marsupials), elephant (eutherian mammals). Organ silhouettes denote the main tissues included, organized by germ layer. **b** Bubble plot showing the number of analyzed samples for each tissue and taxonomic group. **c** Bar plot showing genome-wide DNA methylation levels for each species (black bars outside of the circle), averaged across all tissues and individuals, mapped onto an annotated taxonomic tree. **d** Boxplot showing genome-wide DNA methylation levels for all species, aggregated by taxonomic group. **e** Boxplot showing the percentage of consensus reference fragments for each species that fall into three bins based on their DNA methylation levels, aggregated by taxonomic group. Fragments covered by at least 10 reads were included. **f** Left: Bar plot showing the percentage of variance among species-specific mean DNA methylation levels explained by features of the genomic DNA sequence. Colors indicate the mean Akaike information criterion (AIC), adjusting for model complexity. Error bars represent standard deviations of the mean based on bootstrapping (100 iterations). Right: Bar plot showing the stability with which individual 3-mers were selected into the final model using stepwise selection. Stars indicate that the respective 3-mers show a statistically significant association based on the phylogenetic generalized linear model depicted in panel **h**. **g** Hierarchical clustering of species based on the similarity of their 3-mer and 6-mer frequencies among the consensus reference fragments. Clustering for k-mer lengths of four and five yielded very similar results and is not shown here. The dendrograms are color-coded with each species’ taxonomic group. **h** Scatterplot comparing the statistical significance (*p*-values) of the associations between 3-mer frequencies and global DNA methylation levels based on the standard error of the generalized linear models (GLMs) with (*x*-axis) and without (*y*-axis) correction for phylogenetic relationships. The 3-mers from panel **f** are shown in bold. Dashed lines correspond to an adjusted *p*-value of 0.05. **i** Scatterplot showing the relationship between genome-wide DNA methylation levels and DNA methylation erosion as measured by the “proportion of discordant reads” (PDR) for individual samples. The dashed line represents their mathematically expected relationship. The solid line represents a generalized additive model fitted to the data using the R function *geom_smooth*. **j** Scatterplot showing the relationship between genome-wide DNA methylation levels and DNA methylation erosion across taxonomic groups, taking the median of the corresponding samples. The dashed line represents their mathematically expected relationship (as in panel **i**). The solid line represents a linear regression model fitted to the data. The Pearson correlation and its significance (two-sided) are indicated. **k** Boxplot showing log-ratios of non-CpG methylation levels in brain compared to other tissues in the same species. Boxplots are overlayed with individual data points using the species abbreviations (Supplementary Data 2). Increased non-CpG methylation levels in brain were assessed with a one-sided paired Wilcoxon test. Boxplots are specified as follows: center line, median; box limits, upper and lower quartiles; whiskers, 1.5x interquartile range; points, outliers.

To investigate the evolutionary dynamics of DNA methylation in vertebrates, we performed genome-scale DNA methylation profiling for 580 species and a total of 2443 primary samples (Fig. 1a–c, Supplementary Fig. 1a–f). Our sample collection included all vertebrate classes, and several classes of marine invertebrates, many of them closely related to vertebrates (used here as an outgroup). Specifically, we analyzed samples of 156 invertebrates, one jawless vertebrate (Japanese lamprey, *Lethenteron camtschaticum*), 32 cartilaginous fish (*chondrichthyes*), 565 bony fish (*actinopteri*), 74 amphibians (*amphibia*), 280 reptiles (*reptilia*), 607 birds (*aves*), 70 metatherian mammals / marsupials (*marsupialia*), and 658 eutherian mammals (*mammalia*). Wherever possible, we included multiple tissues (most notably heart and liver for comparison across species) and multiple individuals, with a balanced sex ratio and a focus on young adult animals.

DNA methylation profiling was performed using reduced representation bisulfite sequencing (RRBS). The RRBS assay provides single-nucleotide, single-allele resolution for a defined set of genomic regions determined by sequence-specific DNA fragmentation and size selection. This focus on DNA fragments with defined start and end sequences facilitates DNA methylation analysis across species and without reference genomes (which are unavailable or of inconsistent quality for most analyzed species). RRBS leverages the concept of reduced representation sequencing (also known as RADseq or GBS), a widely used method for genotyping in species that lack high-quality reference genomes^49^. In RRBS, restriction enzymes cut the DNA at CCGG (MspI) and TCGA (TaqI) sites independent of CpG methylation, followed by DNA methylation profiling of the size-selected fragments using bisulfite sequencing. RRBS selects for DNA fragments that each contain at least one CpG, making the assay cost-effective and quantitative even for large genomes with variable CpG density. RRBS covers around four million out of 28 million CpGs in the human genome and two million out of 20 million CpGs in the mouse genome — including CpG-rich promoter and enhancer regions as well as a broad sampling of regions with modest CpG density^50–52^.

We do not expect that differences in genome sequence composition across species will unduly confound the RRBS profiling. MspI and TaqI have short and highly common target sequences, and reduced representation sequencing has been applied successfully in various species^49^. To provide additional support for the validity of using RRBS for cross-species analysis, we simulated RRBS coverage across a wide range of species and analyzed the expected RRBS coverage for genomic elements such as CpG islands, transcripts, promoters, and repetitive elements. Across 76 species for which reference genomes were available (five invertebrates, one jawless vertebrate, one cartilaginous fish, eight bony fish, three amphibian, four reptiles, seven birds, three marsupial, and 44 eutherian mammals), we simulated the restriction digest and size selection in RRBS^53^, and we determined the expected coverage for the different types of genomic elements (Supplementary Fig. 1b). This analysis confirmed that RRBS consistently enriches for CpG islands across all species, and that the observed differences in genomic coverage were small, especially among species of the same taxonomic group.

Because RRBS fragments start and end at defined restriction sites, we do not depend on a reference genome nor on de novo assembly of sequencing reads. Instead, we can group and overlay sequencing reads obtained from the same genomic positions to construct “consensus reference fragments”. We have previously developed and extensively validated the RefFreeDMA method for RRBS-based, reference-genome independent analysis of DNA methylation^45^. Using RefFreeDMA, we can combine the RRBS reads for each species into locus-specific consensus sequences with reconstructed genomic cytosines as the sites of potential DNA methylation. The resulting “consensus reference fragments” were used as the genomic reference for subsequent RRBS read alignment and DNA methylation calling, which was done separately for each sample. To be able to detect constitutively unmethylated cytosines (which appear as thymines in the RRBS reads), we also sequenced one RRBS library without bisulfite conversion for each species and included these data in the identification of genomic cytosines. RRBS quality metrics (such as the number of covered CpGs, mapping efficiency, DNA pre-fragmentation, contamination rate, conversion rate) indicated high data quality for most samples and allowed us to flag potentially problematic samples (Supplementary Fig. 1g, h**;** Supplementary Data 1).

For additional validation, we assessed potential effects of repetitive elements, PCR amplification, and inter-individual genetic variation on our reference-free analysis (Supplementary Fig. 2a). In each species, we empirically flagged consensus reference fragments with consistently high coverage (fourfold above average in >80% of samples) as likely derived from repetitive regions (“repeat”); those with sporadically high coverage (fourfold above average in <20% of samples) as likely subject to PCR amplification biases (“amplified”); and fragments with adequate coverage (at least half of the average) in samples from one individual but not the other individuals as likely results of genetic variation affecting the RRBS coverage (“private”). We found that the frequency of “repeat” and “amplified” fragments was generally low (below 2%) and similar across taxonomic groups, with a trend toward a lower fraction of “repeat” fragments in birds, marsupials, and mammals (Supplementary Fig. 2b, c). We did not observe systematic effects of different PCR cycles across samples, confirming that the range of PCR cycles used (6–18) did not induce strong amplification biases (Supplementary Fig. 2d). Inter-individual genetic variation affected around 10% of the consensus reference fragments, which underlines the importance of investigating several individuals per species.

Finally, for a subset of the analyzed species we can exploit existing reference genomes of the same or related species by cross-mapping of the consensus reference fragments (Supplementary Fig. 3a). We pursued a data-driven approach by mapping the consensus reference fragments of all species to all reference genomes of animals within the same class, selecting the one with highest mapping rate for cross-mapping analysis (Supplementary Fig. 3b, c). This analysis identified the expected association of DNA methylation levels with gene annotations across all taxonomic groups. For example, we detected the characteristic “dip” of DNA methylation in promoter regions (Supplementary Fig. 3d, e) even for unusual species such as the Mexican axolotl with its very large 32-gigabase genome (Supplementary Fig. 3f). We further observed that the strength of the “dip” increased with higher cross-mapping efficiencies up to a rate of 25%, after which this trend leveled off (Supplementary Fig. 3g). In aggregate, these results support that our experimental and bioinformatic methods for comparative analysis of DNA methylation are technically feasible and broadly applicable across species and taxonomic groups.

### Patterns of genome-wide DNA methylation in vertebrate evolution

Having established the validity of our dataset and analysis method, we proceeded with a systematic analysis of factors that predict genome-wide DNA methylation in the analyzed species. We calculated genome-wide DNA methylation levels for each species by averaging across consensus reference fragments, tissues, and individuals, and we overlaid these species-specific aggregates with the taxonomic tree (Fig. 1c). These values provide an assessment of DNA methylation in those genomic regions that RRBS enriches for, such as CpG islands, promoters, and other regulatory elements. They are different from the global DNA methylation content of a sample (i.e., its total 5-methyl-cytosine level), which can be determined biochemically using high performance liquid chromatography (HPLC) and which is usually dominated by repetitive genomic regions^54^.

We observed lower DNA methylation levels in invertebrates compared to vertebrates, lower DNA methylation levels in birds and marsupials than in other vertebrates, and higher DNA methylation levels in fish and amphibia than in other taxonomic groups (Fig. 1d). These trends were driven by differences in the fraction of highly (>80%) versus lowly (<20%) methylated fragments, while fragments with intermediate DNA methylation were similarly common across vertebrate classes (Fig. 1e). These observations were robust across all investigated tissue types (Supplementary Fig. 4a) and across a wide range of technical stringency thresholds (Supplementary Fig. 4b). We also validated our analysis with an independent WGBS dataset comprising 13 species that we curated from the literature^25,55–66^, and we observed a correlation of 0.84 for genome-wide DNA methylation levels based on RRBS versus WGBS (Supplementary Fig. 4c).

Focusing on individual taxonomic groups, in mammalian species, we observed strikingly lower DNA methylation for the two marsupial orders (diprotodontia and dasyuromorpha) compared to other eutherian mammals (Supplementary Fig. 4d). The different groups of reptiles (lizards, snakes, turtles, crocodiles) showed similar levels of DNA methylation—with the exception of *henophidia* (a suborder of snakes including pythons and boas), which had consistently lower DNA methylation levels (Supplementary Fig. 4e). Among the invertebrates—by far the most heterogeneous group in our analysis—we observed a wide range of DNA methylation levels from 2% in *Penaeus* (prawns) to 80% in the clam worm *Alitta succinea*. The majority of these invertebrates had DNA methylation levels in the range of 20–40%, similar to the levels observed in birds and marsupials. Finally, the Japanese lamprey, which is an early jawless vertebrate, showed high DNA methylation levels close to 60%, which is similar to the levels observed in reptiles and mammals (Supplementary Fig. 4f, g).

To quantify and compare the relationship between genome-wide DNA methylation levels and the corresponding genomes, we constructed linear models based on features that describe each species’ DNA sequence composition (e.g., k-mer frequencies, CG composition, CpG island frequency). Strikingly, 3-mer frequencies explained >80% of the observed variance in genome-wide DNA methylation across vertebrate evolution (Fig. 1f). Four of the five most consistent 3-mers contained a CpG dinucleotide (ACG, CGT, CGA, TCG), while the fifth (GAA) has been implicated in mammalian-specific repeat expansions^67^. In contrast, CpG island frequency alone explained only around 23% of the observed variance, and CG composition (with separate variables for C, G, and CpG frequency, as well as the CpG observed versus expected ratio) explained around 50%. These results show that CpG density is a key contributor but clearly not the only factor explaining the close association between genome-wide DNA methylation and genome sequence composition across species.

Similarities in the frequency of short DNA motifs (illustrated here by 3-mers and 6-mers) in the consensus reference fragments of two species closely reflected their phylogenetic distance (Fig. 1g). This observation prompted the question whether 3-mer frequencies predict genome-wide DNA methylation levels directly or through their association with phylogenetic distance. We thus compared the predictive power of 3-mer frequencies with that of phylogenetic distance, which we modeled either by a representation of the taxonomic tree or by assigning each species to its corresponding taxonomic group (Supplementary Fig. 4h). In this analysis, predictions based on 3-mer frequencies (accuracy: 86.4%, Akaike information criterion (AIC): 3819) outperformed those based on either the taxonomic tree (81.0%, 3622) or on taxonomic groups (74.1%, 4154). Nevertheless, combining 3-mer frequencies with phylogenetic information led to a modest increase in overall prediction performance for both taxonomic tree (87.8%, 3371) and taxonomic groups (92.0%, 3514). We further validated our results with generalized linear models that explicitly control for phylogenetic relatedness, and we found that around one third of all 3-mers (22 out of 64) showed a significant association with DNA methylation that was not explained by phylogeny alone (Fig. 1h). These results support that 3-mer frequencies are directly predictive of genome-wide DNA methylation levels, beyond the strong link between phylogeny and 3-mer frequencies.

We also used our dataset to investigate DNA methylation stability and erosion in a wide range of species, motivated by studies that linked DNA methylation erosion to human cancers and ageing^68,69^. We can quantify DNA methylation erosion based on our RRBS data using the “proportion of discordant reads” (PDR) metric^68^. This metric exploits the observation that most genomic loci exhibit a bimodal distribution of DNA methylation (i.e., a locus is either fully methylated or fully unmethylated), and it interprets deviations from this pattern as evidence of DNA methylation erosion. The PDR metric was first established for cancer, where it was associated with clinical features including tumor aggressiveness^68,70–72^. We calculated species-specific PDR values in analogy with the species-specific DNA methylation levels by averaging across consensus reference fragments, tissues, and individuals (Supplementary Fig. 5a). To assess their relationship across species, we plotted genome-wide PDRs over the corresponding species’ genome-wide DNA methylation levels (Fig. 1i, j). Mathematically, we would expect highest PDR values for genome-wide DNA methylation levels around 50% and lower PDR values for higher and lower levels of genome-wide DNA methylation, given the properties of the PDR metric. However, we found that DNA methylation levels of around 75% corresponded to the highest PDR values (Fig. 1i) and that this shift was mainly driven by the taxonomic groups with high DNA methylation levels (amphibians, bony fish) and by reptiles (Fig. 1j). We thus found that these taxonomic groups exhibit unexpectedly high levels of DNA methylation erosion, possibly as a consequence of high genome-wide DNA methylation levels being harder to maintain. In contrast, mammals, birds, marsupials, and cartilaginous fish on average showed slightly lower-than-expected levels of DNA methylation erosion (Fig. 1j), possibly due to molecular mechanisms that foster DNA methylation maintenance in these groups.

We also investigated non-CpG methylation, which is accurately measured by RRBS as shown previously^73^. We found that non-CpG methylation was expectedly low (<2%) but detectable in most samples. We did not observe strong differences in non-CpG methylation across taxonomic groups—with one exception: Brain samples of birds and mammals had elevated levels of non-CpG methylation (Supplementary Fig. 5b), which were on average 59% higher in birds and 72% higher in mammals compared to other organs (Fig. 1k); in contrast, the difference was much weaker for the other taxonomic groups (3% higher in brain than in other organs). Widespread non-CpG methylation in brain samples has been reported previously for both human and mouse^74^ and has been explained by incomplete CpG specificity of mammalian DNA methyltransferases^73^. Our results suggest that this phenomenon generalizes to other mammals and birds, but is not shared by all vertebrates, which may point to differences in the DNA methylation machinery across taxonomic groups^25^.

For vertebrates, it is well-established that DNA methylation levels are high throughout the genome, except for CpG islands, promoters, and other regulatory regions; in contrast, invertebrates are thought to carry a mosaic of high methylation (often at genes) and low methylation (for the bulk of the genome)^75^, although some highly methylated invertebrate genomes have been described^76,77^. In our dataset, we observed pronounced differences when comparing vertebrates and invertebrates as groups—for genome-wide DNA methylation levels at CpGs, for DNA methylation erosion, and to a lesser degree also for non-CpG methylation (Supplementary Figs. 4a, 5a, b). However, these differences were gradual, with overlapping distributions between vertebrate and invertebrate species. In these comparisons, the Japanese lamprey as a jawless vertebrate clearly sided with the vertebrates (Supplementary Fig. 5c).

Finally, we investigated potential associations between DNA methylation and cancer risk, as it had been suggested previously that DNA methylation could be one of many factors that contribute to cancer prevention in large, long-lived species^78^—for example by suppressing repetitive DNA elements that threaten genome integrity or by constraining developmental plasticity in differentiated cells. We observed a positive correlation between genome-wide DNA methylation levels and the theoretical, unmitigated cancer risk of the investigated species (which we estimated based on each species’ body weight and longevity^79^). This positive association was most pronounced in birds (*r* = 0.53) and remained statistically significant after correcting for phylogeny (*p* = 0.01) (Supplementary Fig. 5d). We also observed similar but weaker associations for DNA methylation erosion (likely due to positive correlation between DNA methylation and PDR) but not for non-CpG methylation. While these observations do not imply a specific causal role of DNA methylation on cancer risk, they contribute to accumulating evidence of associations between DNA methylation and cancer risk across species.

### A genomic code for DNA methylation in vertebrates and invertebrates

While the previous section focused on genome-wide measures of DNA methylation across species, our dataset also allows us to investigate locus-specific DNA methylation levels within each analyzed species, pursuing the hypothesis that there is a predictive relationship (or “genomic code”) between the DNA sequence of a given genomic region and its DNA methylation level. Previous studies in human and mouse have uncovered associations between DNA methylation and the underlying DNA sequence for genomic regions such as CpG islands and gene promoters^38,39^ and for single CpG dinucleotides throughout the genome^40,41^; and it is possible that the genomic DNA sequence encodes a default epigenetic “ground state” for each genomic region, to which its DNA methylation is reset in early embryonic development^80^. Nevertheless, here we use the term “genomic code” solely as a shorthand for a predictive relationship between DNA sequence and DNA methylation, without implying causality or proposing any specific molecular mechanism that would read this code.

**Fig. 2:**
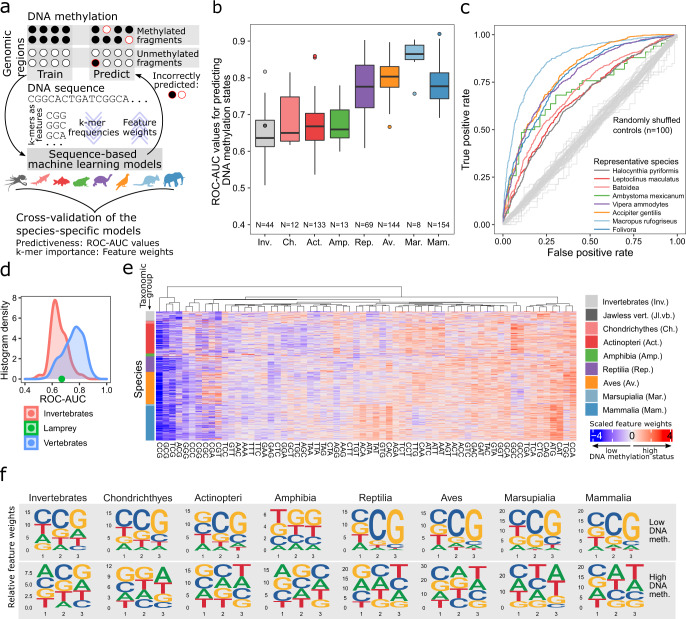
Machine learning identifies a predictive relationship (“genomic code”) between the DNA sequence and locus-specific DNA methylation. **a** Schematic illustration of the machine learning based approach for predicting locus-specific DNA methylation from the underlying genomic DNA sequence. **b** Boxplot showing the test set performance (receiver operating characteristic area under curve, ROC-AUC) of support vector machines (SVMs) predicting the DNA methylation status (high versus low) of individual genomic regions based on the k-mer frequencies of the corresponding genomic DNA sequence. **c** Representative ROC curves for each taxonomic group, selected such that the displayed species’ ROC-AUC values closely reflect the mean ROC-AUC values of the corresponding taxonomic group. As negative controls, ROC curves trained and evaluated on data with randomly shuffled labels fall close to the diagonal (in gray). **d** Histograms of ROC-AUC values for vertebrate and invertebrate species, with the lamprey (an early jawless vertebrate) shown as a green dot between the two distributions. **e** Heatmap displaying the feature weights of 3-mers based on SVMs trained to predict locus-specific DNA methylation from the underlying DNA sequence, separately for each species (ordered by the taxonomic tree). **f** Sequence logos visualizing averaged feature weights of 3-mers across species for each taxonomic group. Sequence logos are displayed separately for 3-mers associated with low and high DNA methylation levels. Boxplots are specified as follows: center line, median; box limits, upper and lower quartiles; whiskers, 1.5x interquartile range; points, outliers.

To decipher the relationship between DNA methylation and the underlying DNA sequence throughout vertebrate evolution, we trained machine learning classifiers that predict locus-specific DNA methylation levels based on the DNA sequence of the corresponding genomic regions (Fig. 2a). Specifically, we used support vector machines with a spectrum kernel to predict the discretized DNA methylation status (highly versus lowly methylated) of consensus reference fragments based on their genomic DNA sequence (represented by k-mer frequencies), separately for each species. The prediction performance was quantified using receiver operating characteristic (ROC) curves and area under curve (AUC) values calculated on independent test sets. The robustness of these predictions was confirmed using two alternative definitions of methylated and unmethylated regions with different stringency, which resulted in highly similar ROC-AUC values (Supplementary Fig. 6a).

We consistently observed greater-than-random prediction performance across all taxonomic groups (Fig. 2b, c), with higher mean ROC-AUC values in reptiles, birds, and mammals (0.78, 0.80, 0.78) than in cartilaginous fish, bony fish, and amphibians (0.68, 0.67, 0.70). The prediction performance was markedly higher in marsupials (0.86) compared to eutherian mammals (0.78), indicating that DNA methylation may have a particularly pronounced genetic basis in marsupials. For invertebrates (0.65), the prediction performance was slightly lower than that of fish. The lamprey (0.67), an early jawless vertebrate, fell in between vertebrates and invertebrates (Fig. 2d), which adds to the evidence that differences in DNA methylation between vertebrates and invertebrates are more gradual and less pronounced than DNA methylation differences between animals and plants^81^. Overall, our results support the existence of a “genomic code” that links locus-specific DNA methylation levels to the underlying DNA sequence in vertebrate and invertebrate species.

To dissect this predictive relationship, we compared the cross-validated prediction performance of classifiers trained on 1-mer frequencies (A, C, T, G), 2-mer frequencies, etc. up to 10-mer frequencies. In this analysis, 3-mer frequencies were generally the most informative, followed by 2-mer and 4-mer frequencies (Supplementary Fig. 6b, c). In contrast, the inclusion of longer DNA sequence patterns did not result in greater predictive power, suggesting that complex motifs (including those that correspond to transcription factor binding sites) are less relevant for the association between DNA methylation and DNA sequence than short sequence patterns. We independently validated our trained models by testing them on DNA methylation profiles obtained by reference-based analysis of publicly available WGBS data for eight species, and we also performed the inverse analysis—training on WGBS data and testing on our RRBS data (Supplementary Fig. 6d). Moreover, we obtained highly consistent results including similar ROC-AUC values and preferred k-mer lengths between purely RRBS-based and purely WGBS-based predictions (Supplementary Fig. 6e).

Finally, we inferred the predictive power of individual 3-mer frequencies for each species, and we compared the corresponding weights across all taxonomic groups (Fig. 2e, f; Supplementary Fig. 6f). 3-mers associated with low DNA methylation levels preferentially ended with CpG dinucleotides and started with either a C or G nucleotide. This pattern was conserved across all taxonomic groups, including invertebrates; but it was more pronounced in reptiles, birds, marsupials, and eutherian mammals compared to invertebrates, fish, and amphibians. In contrast, 3-mers associated with high DNA methylation levels followed a more diverse DNA sequence composition, and the enrichment for specific DNA sequence patterns differed between taxonomic groups. Most notably, invertebrates showed an enrichment of CpG dinucleotides also among highly methylated regions, which distinguished them from vertebrates; and mammals showed an enrichment of CpA dinucleotides, which tend to arise from preferential CG to TG/CA mutations for methylated CpG dinucleotides^82^.

### Conservation and divergence of the genomic code for DNA methylation

**Fig. 3:**
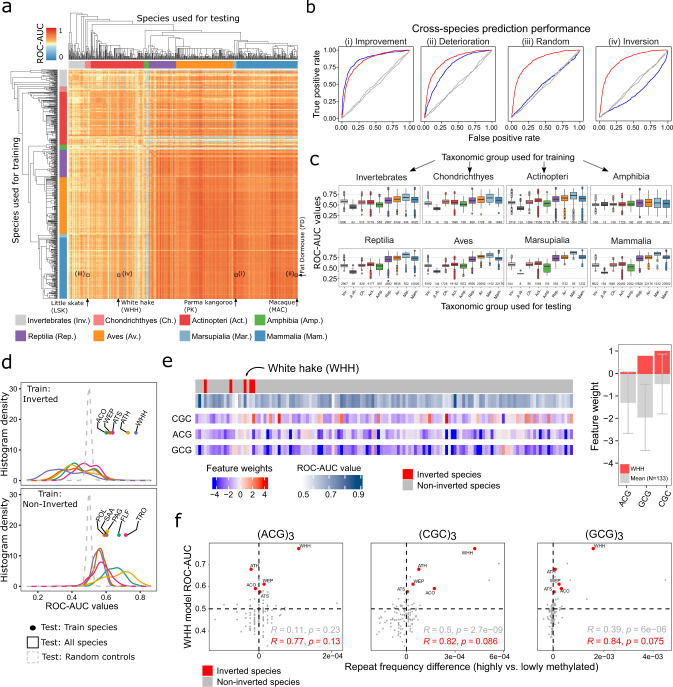
The “genomic code” of locus-specific DNA methylation is broadly conserved across vertebrate and invertebrate species. **a** Heatmap showing ROC-AUC values for the prediction of locus-specific DNA methylation from the underlying DNA sequence between all pairs of species. The comparisons of panel **b** are highlighted in the heatmap. **b** ROC curves illustrating characteristic outcomes of cross-species predictions, for classifiers trained in one species (fat dormouse, abbreviated as FD) and tested in other species (left to right: Parma wallaby, PK; macaque, MAC; little skate, LSK; white hake, WHH). “Inverted species” are characterized by worse-than-random prediction performance when training is performed in a non-inverted species. **c** Boxplots summarizing the cross-species prediction performance (i.e., ROC-AUC values from panel **a**) aggregated by taxonomic group of the training species (individual plots) and test species (*x*-axis). **d** Histograms of cross-species prediction performance (i.e., ROC-AUC values from panel **a**) for all inverted fish species (top) in comparison to phylogenetically related non-inverted species (bottom). Inverted species: Atlantic cod, ACO; walleye pollock, WEP; Atlantic salmon, ATS; Atlantic herring, ATH; white hake, WHH. Non-inverted species: Pollock, POL; silver arowana, SAA; Pacific grenadier, PAG; onefin flashlightfish, FLF; trout, TRO. **e** Left: Heatmap showing classifier feature weights for the most differential 3-mers between an inverted species (white hake, WHH) and all other bony fish (*actinopteri*) species, ordered by the taxonomic tree. Right: Bar plots for the weights of the same 3-mers in white hake compared to their average across all other bony fish (*actinopteri*) species. Error bars indicate standard deviations of the mean. **f** Scatterplots for the association between the cross-species prediction performance (*y*-axis) of classifiers trained in an inverted species (white hake, WHH) and the difference in frequency of three 9-mer repeats (*x*-axis) constructed by the repetition of the differentially weighted 3-mers from panel **d**. Values above 0 indicate higher frequency in highly methylated sequences and vice versa. The following inverted species are shown: Atlantic cod (ACO), walleye pollock (WEP), white hake (WHH), Atlantic salmon (ATS), Atlantic herring (ATH). Dashed lines indicate a frequency difference of 0 (vertical line) and a ROC-AUC value of 0.5 (horizontal line). The Pearson correlation and its significance (two-sided) are indicated. Boxplots are specified as follows: center line, median; box limits, upper and lower quartiles; whiskers, 1.5x interquartile range; points, outliers.

Our results support the existence of a “genomic code”—a predictive relationship between DNA sequence and DNA methylation with differential 3-mer frequencies of methylated and unmethylated loci. While this association between the genome and epigenome was generally consistent across all investigated taxonomic groups, we also observed characteristic differences, both between taxonomic groups and between individual species within a group. To investigate these evolutionary differences more systematically, we trained machine learning models in one species and applied them (without retraining) to predict DNA methylation levels in another species. For each pair of species, we then determined ROC-AUC values as measures of cross-species predictability, with high values indicating good transferability of the trained model from one species to the other (Fig. 3a, b).

The prediction performance was generally high between related species and even across taxonomy groups (Fig. 3c), often reaching similarly high values as for the prediction within a species or within a taxonomy group. However, we also observed pronounced differences in cross-species predictability, notably between invertebrates, fish, and amphibians on the one hand (where the predictability was lower) and the evolutionarily younger groups of reptiles, birds, and mammals on the other hand (where the predictability was higher).

We found that models trained in species with lower prediction performance generally performed well in species with higher prediction performance, but not vice versa (Fig. 3a–c). Even prediction models trained in invertebrates retained some predictive power in vertebrates, despite previously described differences in the genomic distribution of DNA methylation between vertebrate and invertebrate genomes^75^. These observations suggest that the predictive relationship between locus-specific 3-mer frequencies and their associated DNA methylation levels (i.e., the “genomic code” of DNA methylation) is deeply conserved across taxonomic groups. However, the fact that predictability of DNA methylation differed widely across target species suggests that some species deviate much more strongly from the genetically encoded “default” DNA methylation profile than other species, perhaps due to tissue-specific regulation, environmental influences, and stochastic effects.

Curiously, a few species of invertebrates, fish, amphibians, and reptiles showed an apparent inversion of the genomic code for DNA methylation, such that DNA sequence patterns normally associated with low DNA methylation levels were instead linked to high DNA methylation levels, and vice versa. The presence of such inverted species in our dataset was evident from ROC-AUC values for cross-species prediction that were substantially worse than expected by random chance (blue horizontal/vertical stripes in Fig. 3a; example shown in 3b). In other words, prediction models trained in non-inverted species and applied to the inverted species misclassified methylated regions as unmethylated, and unmethylated regions as methylated, at a frequency that could not be explained by random chance. We observed the same pattern of significantly lower-than-random prediction performance when models were trained in the inverted species and applied in non-inverted species. In contrast, cross-species prediction between two inverted species gave more consistent results than prediction between one inverted and one non-inverted species (Supplementary Fig. 7a), suggesting shared patterns among the inverted species.

To investigate the biological basis of this apparent inversion of the genomic code for DNA methylation, we focused on the white hake (*Urophycis tenuis*), a bony fish (*actinopteri*) species with pronounced inversion (Fig. 3d) that was consistently detected across tissues and individuals (Supplementary Fig. 7b–f). When we compared the feature weights of the trained classifiers for predicting DNA methylation, we identified three 3-mers (ACG, CGC, GCG) that were strongly predictive of high DNA methylation levels in white hake but predictive of low DNA methylation levels in other bony fish (Fig. 3e). These 3-mers were associated with highly methylated repetitive elements in the white hake and to a lesser degree also in other inverted fish species, but not in most of the non-inverted fish species (Fig. 3f). We tentatively conclude that the observed inversion may be explained by introgression of evolutionarily recent, CpG-rich, repetitive elements, which often acquire high DNA methylation levels due to the cells’ machinery for suppressing their genomic instability and expansion^1^.

### Evolutionary conservation of tissue-specific DNA methylation patterns

**Fig. 4:**
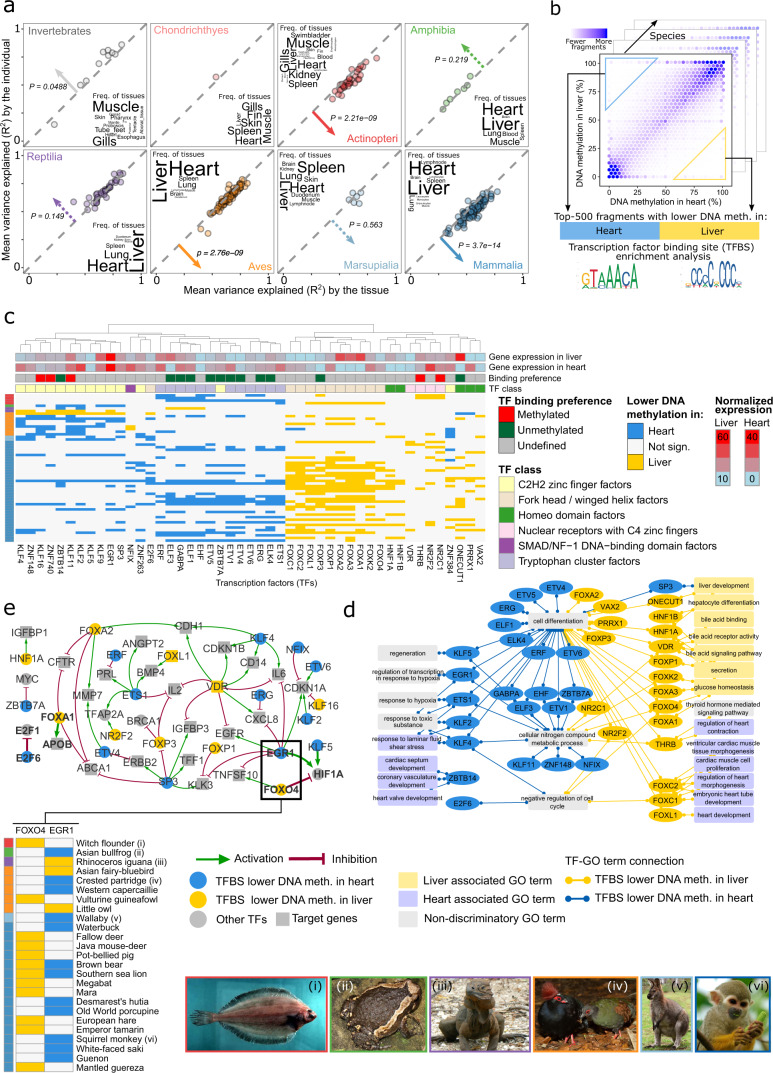
Tissue-specific DNA methylation indicates deeply conserved associations of DNA methylation with transcription regulation and tissue identity. **a** Scatterplots showing for each species the percentage of locus-specific DNA methylation variance that is explained by the tissue (*x*-axis) and by the individual (*y*-axis), separately for each taxonomic group. Arrows and *p*-values indicate the direction and statistical significance of the difference in the variance explained by tissue and individual, calculated using a two-sided pairwise Wilcoxon test. Dashed arrows indicate non-significant differences. Word clouds summarize the frequency of tissue types that contributed to the analysis in each taxonomic group. **b** Schematic illustration of the enrichment analysis for transcription factor binding site (TFBS) motifs among the differentially methylated regions identified between heart and liver (within a given species). **c** Clustered heatmap showing TFBS motif enrichments for differentially methylated fragments between heart and liver. For each transcription factor (columns), colors indicate whether it was enriched in fragments that were hypomethylated in heart (blue) or liver (yellow) in the corresponding species (rows). This heatmap includes only those transcription factors and species that had a minimum of ten significant enrichments per species, and normalized RNA expression values greater than one in either heart or liver tissues according to the Human Protein Atlas. **d** Visualization of the Gene Ontology annotations of the transcription factors identified in panel **c**. **e** Gene-regulatory network constructed based on the transcription factors identified in panel **c** with known binding preference (methylated/unmethylated) and their direct target genes with known regulatory effect (activation: green; repression: red). Transcription factors that were preferentially hypomethylated in one tissue type were colored in yellow (heart) or blue (liver), while those that did not show such an enrichment, as well as the transcription factor target genes, were colored in gray. The inset shows specific enrichments for FOXO4 and EGR1 in heart and liver, which have opposing effects on HIF1A (FOXO4: activation; EGR1: repression). The pictures at the bottom show one species for each taxonomic group that contributed to this cross-species analysis of DNA methylation differences in heart and liver.

The hypermethylation of repetitive elements in the inverted species (as described in the previous section) appears to occur on top of a broadly conserved “genomic code” of DNA methylation. We would expect that the same applies to tissue-specific as well as inter-individual differences in DNA methylation. To investigate the relative contributions of the tissue and the individual to the observed DNA methylation variation in our dataset, we focused on those species for which we have multiple tissues and individuals (*n* = 360). For each of these species we inferred the percentage of variance explained by the tissue and by the individual (Fig. 4a).

In human and mouse, it is well established that DNA methylation patterns are more similar among samples of one tissue from different individuals than among samples of different tissues from one individual^44,83,84^. We observed this phenomenon for a subset of species from all taxonomic groups except cartilaginous fish, which are not sufficiently covered for a confident assessment (Supplementary Fig. 8a). However, when quantifying the influence of these factors across species, we found that tissue-specific differences clearly exceeded inter-individual differences only in mammals, birds, and bony fish, whereas we observed equal or higher variability explained by the individual than by the tissue for many invertebrates, amphibians, and reptiles (Fig. 4a).

These different contributions of tissue-specific and individual-specific factors were not an artifact of differences in technical data quality, as measured by PCR enrichment cycles in the RRBS protocol (Supplementary Fig. 8b) and by DNA pre-fragmentation as a proxy for low DNA quality (Supplementary Fig. 8c). Nor was it explained by differences in genetic variation, which we estimated based on the mean overlap in covered CpGs between samples from the same species (Supplementary Fig. 8d). In contrast, we found a negative correlation between DNA methylation erosion on the one hand and the variance explained by the tissue or individual on the other hand (Supplementary Fig. 8e). This inverse association was observed and statistically significant for reptiles, birds, and mammals. It is indicative of species-specific differences in the share of DNA methylation variation that should be attributed to random fluctuations and unexplained biological noise.

Next, we investigated DNA methylation in its relation to tissue identity, seeking to identify patterns of tissue-specific DNA methylation that are conserved throughout vertebrate evolution. We focused on heart and liver, the two tissues that are best represented in our dataset. We were able to include 207 species in this analysis, for which heart and liver samples were available from at least two individuals each. For each species, we identified differentially methylated consensus reference fragments between heart and liver (Fig. 4b) and compared the enrichment for transcription factor binding motifs between fragments with lower DNA methylation levels in heart versus lower levels liver (Fig. 4c, Supplementary Fig. 9a). This analysis exploits the observation that many transcription factors and their binding motifs are conserved across vast evolutionary distances^85^. We indeed detected many transcription factor binding motifs at similar frequencies in fragments from all taxonomic groups, with no obvious preference for mammals (Supplementary Fig. 9b).

For further biological interpretation, we determined the transcription factors that are expressed in human heart or liver tissues and whose binding sites were enriched in differentially methylated fragments. We further annotated these fragments with GO terms related to heart and liver biology, physiology, and gene regulation (Fig. 4d). We found that transcription factors associated with fragments characterized by lower DNA methylation levels in heart were preferentially annotated with heart-specific biological functions (e.g., ZBTB14 has a role in cardiac septum development; KLF4, KLF2, and ETS1 are involved in the response to laminar fluid shear stress). Conversely, fragments with lower DNA methylation levels in liver were annotated with liver-specific functions (e.g., ONECUT1 and HNF1A contribute to liver development; FOXP1, FOXA1, FOXK2, FOXA3, and FOXO4 are involved in glucose homeostasis). Moreover, the binding sites of several transcription factors with a role in the response to hypoxia and to toxic substances had lower DNA methylation levels in heart than in liver, which may be linked to the liver’s greater tolerance for such exposures. While these results are consistent with the concept that low DNA methylation levels are associated with high regulatory activity^35^, we also found one striking example in which higher DNA methylation levels appear to coincide with higher regulatory activity: Fragments enriched for the binding sites of FOXC2, FOXC1, and FOXL1—three FOX family transcription factors with an established role in heart development—showed lower DNA methylation levels in liver than in heart, indicative of diverse relationships between DNA methylation and regulatory activity^35^.

Finally, we inferred the evolutionarily conserved “tissue of activity” for individual transcription factors based on transcription factor binding site enrichment, while taking into account preferential binding to unmethylated or methylated DNA^86^ (Fig. 4c). From the identified transcription factors we derived a gene-regulatory network using regulator interactions obtained from the TRRUST v2 database^87^ (Fig. 4e). This network constitutes a first exploratory attempt to reconstruct an epigenetic contribution to tissue identity of heart and liver that is deeply conserved across vertebrates. Our analysis indicates that FOXA1 (also known as hepatocyte nuclear factor 3-alpha) is active in liver and may induce APOB, a crucial component of low density lipoprotein (LDL) produced in the liver and small intestine^88^. E2F6, a transcriptional repressor involved in cell cycle regulation^89^, showed higher inferred activity in heart than in liver, potentially reflecting the different regenerative potential of these two organs. HIF1A (hypoxia-inducible factor 1-alpha) may be repressed by high activity of FOXO4 in liver, while being activated by KLF5 and EGR1 in heart, which may contribute to the higher tolerance toward hypoxic conditions in the liver^90^. These observations are in line with our GO analysis (Fig. 4d) and suggest that DNA methylation may help stabilize the fundamental regulatory processes underlying vertebrate tissue identity, in ways that are conserved across large evolutionary distances.

### Gene-centric patterns of DNA methylation in vertebrate evolution

**Fig. 5:**
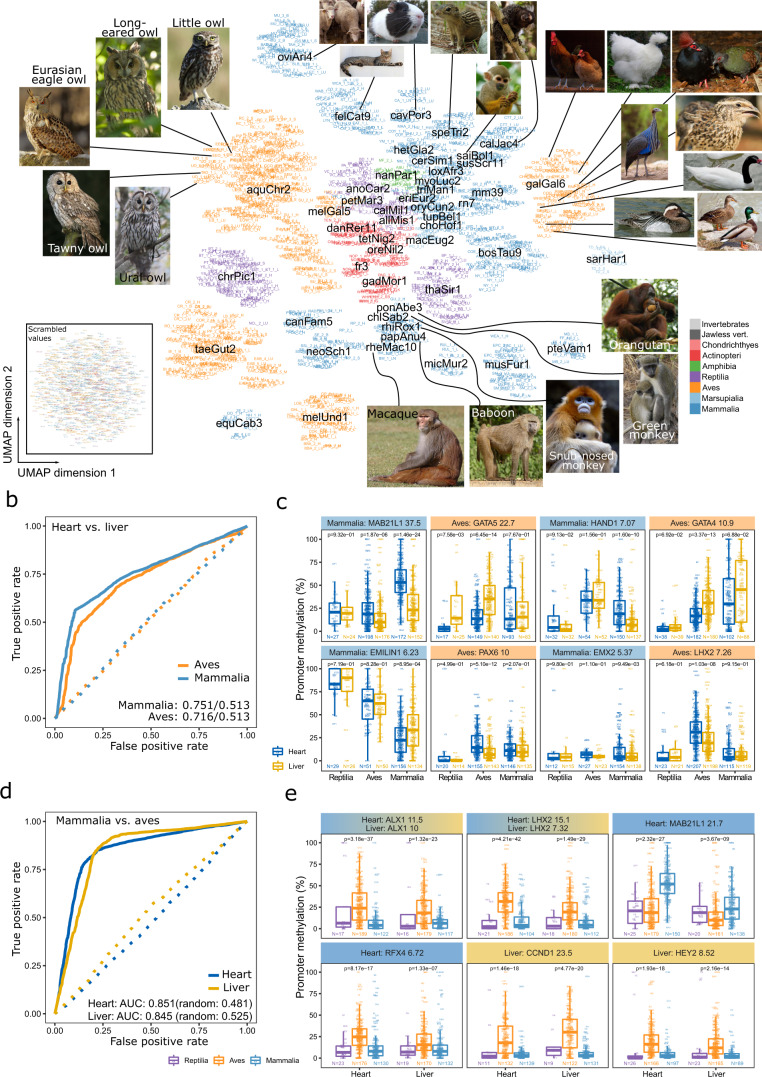
Cross-species analysis of DNA methylation in the human ortholog gene space identifies both conservation and divergence of promoter methylation. **a** UMAP representation of DNA methylation at gene promoters based on cross-mapping of reference-free consensus reference fragments to annotated reference genomes. Samples are colored by taxonomic group, and the matched reference genomes are overlayed in black. Each sample is labeled by its sample identifier (Supplementary Data 1), which is searchable and readable when zooming into the PDF of the figure. Reference genomes are annotated by their UCSC Genome Browser identifiers (e.g., aquChr2 for the golden eagle genome, as described in the Methods section). Inset: UMAP representation of scrambled data, showing the lack of clustering in a control analysis. **b** ROC curves for random forest classifiers using the cross-mapped dataset to distinguish between heart and liver based on promoter methylation data for birds and mammals. The solid lines are based on the actual data, while the dashed lines are based on scrambled data (as in the inset in panel **a**). ROC-AUC values are given for the actual data (first) and scrambled data (second). **c** Boxplots showing DNA methylation levels at gene promoters for the four most predictive genes in the classification of heart versus liver, aggregated by taxonomic groups and overlayed with individual data points using the species abbreviations (Supplementary Data 2). Gene names and the predictiveness (feature importance) of their promoter methylation are indicated in the header bars. *P*-values were calculated using a two-sided Wilcoxon test. **d** ROC curves for random forest classifiers using the cross-mapped dataset to distinguish between birds and mammals based on promoter methylation data for heart and liver samples. The format is identical to panel **b**. **e** Boxplots showing DNA methylation levels at gene promoters for the four most predictive genes in the classification of mammals versus birds. The format is identical to panel **c**. Boxplots are specified as follows: center line, median; box limits, upper and lower quartiles; whiskers, 1.5× interquartile range; points, outliers.

Reference-free analysis of DNA methylation, which has been the basis of all results described in the previous sections, allowed us to include all 580 species, unconstrained by the availability of reference genomes. However, this approach makes it difficult to link DNA methylation patterns to the genes and promoters that they may regulate. We therefore complemented our reference-free analyses with a reference-based analysis of DNA methylation in which we cross-mapped the samples to annotated reference genomes of the same or related species, with data-driven selection of the most fitting reference genome for each species (Supplementary Figs. 3a–c, 10a). We calculated mean DNA methylation levels of individual gene promoters for each sample, based on the gene annotations of the corresponding reference genome. We then linked these annotations to their human ortholog, to analyze gene promoter methylation from all species in one shared gene space (Supplementary Fig. 10a). We thus derived a gene-centric DNA methylation landscape comprising 382 species, 1524 cross-mapped samples, and 14,339 genes, which we projected on two dimensions to visualize relationships in the shared space of DNA methylation at gene promoters (Fig. 5a).

This cross-species landscape of DNA methylation reflects phylogenetic relationships as well as similarities and differences in DNA methylation that are associated with species, tissues, and individuals (Fig. 5a, Supplementary Fig. 10b). The most informative results were obtained for mammals, given the large number of available reference genomes and the conservation of human genes, which ensures an accurate mapping. For example, samples mapped to the reference genomes of old-world monkeys (rhesus, baboon, snub-nosed monkey, green monkey) and apes (orangutang) formed a clear cluster, while samples mapped to the reference genomes of new-world monkeys (marmoset, squirrel monkey) constituted a separate group. Among birds, the golden eagle genome (genome assembly: aquChr2) and the chicken genome (genome assembly: galGal6) enabled gene-centric analyses for multiple other species including owls and ducks, respectively. Fish, amphibians, and reptiles were not as well represented as mammals and birds, but still detectable in this gene-centric analysis, due to high conservation of certain genes over long evolutionary timescales. The observed patterns were clearly non-random and not seen in scrambled data (Fig. 5a, inset).

We exploited this cross-species landscape to define groups of genes that exhibit similar patterns of DNA methylation at their promoters throughout vertebrate evolution. To this end, we projected all adequately covered genes on two dimensions using the UMAP method, and we identified five distinct gene sets using the Leiden clustering method (Supplementary Fig. 10c). Cluster 1 was characterized by high promoter methylation in mammals, and specifically in samples from the lymph node (a tissue that is largely restricted to mammals); Cluster 2 showed consistently low promoter methylation across taxonomic groups and tissues, and was enriched for GO terms related to organ morphogenesis; Cluster 3 exhibited high levels of promoter methylation in birds, and in brain and several internal organs, and it was enriched for GO terms relating to organism development; Cluster 4 was associated with high promoter methylation in reptiles and bony fish but low promoter methylation in cartilaginous fish; Cluster 5 was characterized by low promoter methylation in various internal organs but high promoter methylation in blood, skin, fins, and gonads (Supplementary Fig. 10d, e).

To assess conservation and divergence of tissue-specific DNA methylation for individual gene promoters, we used random forest classifiers to identify genes with tissue-predictive or taxonomy-predictive promoter DNA methylation (Fig. 5b–e). We focused on the two best represented tissues (heart, liver) and taxonomic groups (mammals, birds) and devised four classification tasks: Heart versus liver in each of the two taxonomic groups, and mammals versus birds for each of the two tissues. In these analyses, we ensured that all models were tested only on species that had not been used during training, in order to focus on patterns that are conserved across species. We obtained good prediction performance for all four tasks: ROC-AUC in the heart versus liver classification were 0.751 for mammals and 0.716 for birds, while the corresponding values for the classification of mammals versus birds were 0.851 for heart and 0.845 for liver (Fig. 5b, c).

We investigated which gene promoters support these predictions and found that the most discriminatory genes between heart and liver (Fig. 5c) were transcription factors. This included GATA4 and GATA5 in birds, which have well-known roles in heart differentiation. In mammals, we identified three transcription factors (MAB21L1, HAND1, EMX2) but also the EMILIN1 gene, which codes for a protein that anchors smooth muscle cells to elastic fibers and may be important for heart function. This gene showed increasing promoter methylation from mammals over birds to reptiles, possibly linked to the marked anatomical and functional changes during evolution of the circulatory system. Moreover, MAB21L1, a cell fate regulator with similarity to the cGAS innate immune sensor^91^ displayed higher promoter methylation in heart tissues of both mammals and birds. The most discriminatory genes between birds and mammals (Fig. 5e) were similar between heart and liver tissue; this included the homeobox genes ALX1 and LHX2 as well as the cell cycle promoting cyclin CCND1, all of which had significantly higher promoter methylation levels in birds compared to reptiles and mammals.

Finally, we performed gene-centric analysis of promoter methylation across all eight taxonomic groups (Supplementary Fig. 10f), and we identified 48 genes that had a conserved promoter methylation signal across most of the assessed taxonomic groups. Only one gene retained an unmethylated promoter throughout vertebrate evolution: CHCHD7, a putative housekeeping gene that is ubiquitously expressed in human tissues. In contrast, the promoter of SPON2, which codes for a cell adhesion protein involved in innate immunity, was highly methylated in all classes except marsupials. Genes with high promoter methylation across taxonomic groups (such as SPON2, LMF1, NRDE2, SLC38A10, VASN, NUDT7, GNL2, NETO1, APRT, FAM163B, ALOX5) had a tendency toward higher methylation levels in mammals compared to other taxonomic groups, while most other genes had lower DNA methylation levels in mammals. A similar pattern was observed also for reptiles, while birds and marsupials often showed low promoter methylation levels even for genes with highly methylated promoters in other taxonomic groups. Fish and amphibia had high promoter methylation in most of the 48 broadly conserved genes, consistent with their generally high DNA methylation levels. Lamprey, as a jawless vertebrate, displayed promoter methylation patterns similar to those of cartilaginous fish. In contrast, invertebrates generally had low promoter methylation levels even for genes with high promoter methylation across all other taxonomic groups, supporting diverging gene-regulatory roles of DNA methylation between vertebrates and invertebrates.

Given the breadth of the presented dataset and analysis, detailed follow-up studies in selected species will be needed to corroborate and extend these observations; and we provide our dataset as a comprehensive resource and starting point for such investigations (http://cross-species-methylation.bocklab.org).

## Discussion

We performed a comparative analysis of DNA methylation over the course of vertebrate evolution, based on genome-scale DNA methylation profiles for 2443 tissue samples from 580 animal species. This dataset allowed us to address fundamental biological questions with adequate resolution and statistical power. Most notably, we investigated the relationship of DNA methylation and DNA sequence, the prevalence of DNA methylation erosion, the role of tissue versus individual as sources of DNA methylation variation, and the conservation of gene-regulatory DNA methylation signatures throughout vertebrate evolution.

Our study was enabled by a highly scalable method for DNA methylation profiling and data analysis that is applicable to essentially any species and tissue sample, allowing us to capitalize on large zoological biobanks and sample collections of wild, pet, and zoo animals. The optimized RRBS assay proved robust for samples of variable quantity and quality, consistent with our previous experience working with challenging formalin-fixed paraffin-embedded patient samples^70^. We were thus able to include many samples obtained during routine dissection of deceased animal. Using our RefFreeDMA software, we analyzed and compared DNA methylation independent of whether a reference genome has been established for these species. We extensively validated our DNA methylation profiling and analysis method using coverage simulations across 76 reference genomes and validation of key results with a meta-analysis of WGBS data for 13 species. In addition, we cross-mapped the results of our reference-free analysis to gene-annotated reference genomes, which provided additional validation and gene-centric insights, while also illustrating the future value of our dataset for reference-based analysis, as many more high-quality reference genomes will become available over the next decade.

At the core of our study, we used machine learning to predict DNA methylation levels based on the DNA sequence, with the goal of linking genomes and epigenomes throughout vertebrate evolution. We refer to the predictive relationship between DNA methylation and the underlying DNA sequence as a “genomic code” that connects DNA methylation states to preferred DNA sequence motifs. While we are not implying any specific mechanism or direct causation, we found that this “genomic code” was highly conserved across all analyzed taxonomic groups. Both for genome-wide and locus-specific DNA methylation levels, this relationship was best described by 3-mer frequencies. As expected, high frequency of CpG dinucleotides was associated with low DNA methylation levels, but CpGs were by no means the only contributing factor. Machine learning models trained to predict locus-specific DNA methylation levels from the underlying DNA sequence in one species generally performed well also in other species, even across taxonomic groups, and the prediction performance appeared to be more a feature of the target species than of the species in which the model was trained.

This broadly conserved “genomic code” was detectable even among invertebrate species, to the point that models trained on DNA methylation data for invertebrate species retained some predictive power for vertebrate species. More generally, our dataset uncovered an unexpected degree of conservation in the characteristics of DNA methylation between vertebrates and invertebrates. First, while invertebrates on average showed lower genome-wide DNA methylation levels than vertebrates, many invertebrate species had genome-wide DNA methylation levels well within the distribution of vertebrates (Supplementary Figs. 4f, 5c). Second, certain invertebrate species such as sea urchins (*Strongylocentrotus*) showed DNA methylation profiles that are similar to those of vertebrates, with a prominent dip at gene promoters and high gene-body methylation (Supplementary Fig. 3d, e). Third, the lamprey fell between vertebrates and invertebrates in terms of the predictiveness of the “genomic code” of DNA methylation, consistent with its intermediate position as an early jawless vertebrate.

Considering the full dataset of 580 species, we conclude that the changing characteristics of DNA methylation throughout vertebrate evolution are more gradual and diverse than previously reported based on smaller datasets and widely studied model organisms. Nevertheless, two major transitions in the “genomic code” of DNA methylation are supported by our study. These two transitions are associated with the emergence of vertebrates and with the emergence of reptiles, respectively. They manifested themselves by more accurate predictions of DNA methylation from DNA sequence for reptiles, birds, marsupials, and mammals than for fish and amphibians, and by characteristic similarities and differences in the most predictive DNA sequence motifs (Fig. 2).

While we cannot claim any firm causal or mechanistic insights based on our observational data, we speculate that the predictive “genomic code” of DNA methylation may play a role in the faithful restoration of default DNA methylation patterns—not only in embryogenesis and germ cell development^80^, but also following artificial DNA methylation depletion^92,93^. A default epigenetic state that is encoded in the DNA sequence may provide the baseline that is modulated over a lifetime by other effects such as cell differentiation, environmental exposures, organismal ageing, and random chance. To understand how such a default epigenetic state is established and why it differs across taxonomic groups, it will be interesting to combine our cross-species data with an investigation of the biochemical machinery that controls DNA methylation—including DNA methyltransferases^13^ and demethylases^94^, but also histone-modifying enzymes^95^ and transcription factors that affect DNA methylation.

A side note of our study is the cross-species analysis of DNA methylation erosion, which uncovered pronounced erosion in the taxonomic groups with high levels of DNA methylation (amphibians and fish), rather than in taxonomic groups with intermediate levels (mammals and reptiles) as it would be expected mathematically^68^. This observation may be due to higher DNA methylation levels being intrinsically harder to maintain, given the limited fidelity of maintenance DNA methylation^96^. The affected species would therefore need to tolerate lower stability of DNA methylation, with the potential upside of creating more room for accommodating environmental and stochastic influences on DNA methylation.

Erosion of DNA methylation patterns has been observed in human cancers^68,70–72^, and loss of epigenetic control appears to causally contribute to cancer development^8,34,97^. In this context, the observed variability of DNA methylation erosion across species raises interesting questions regarding a potential role of DNA methylation as a cancer-protective mechanism, especially in large and long-lived vertebrates. While our dataset cannot functionally address these questions, we found that birds, which have a low incidence of tumors^98^, were characterized by particularly low levels of DNA methylation erosion and showed a positive correlation between theoretical cancer risk and DNA methylation levels. We envision that our optimized RRBS assay and reference-free analysis will facilitate DNA methylation profiling of tumors from wild and zoo animals for the wide range of vertebrate species encountered in veterinary pathology. This will in time contribute to a better understanding of the potential roles of DNA methylation in solving the lack of correlation between body size and cancer risk (Peto’s paradox)^99,100^, which stands out as a remarkable feat of vertebrate evolution.

Potential limitations of this study arise from the experimental choices that allowed us to process 2443 primary tissue samples from 580 species. First, RRBS uses restriction enzymes to pre-enrich a “reduced representation” of the genome prior to bisulfite conversion and sequencing. Compared to WGBS, RRBS covers fewer CpGs (mean: 2.5 million CpGs per sample), is cheaper, and more scalable. It also provides consistent start and end points for the DNA fragments defined by the restriction sites, which facilitates the comparison across tissues and across individuals (mean: 1.7 million shared CpGs between two samples of the same species). Second, analyzing any subset of CpGs in the genome bears the risk of introducing species-specific biases. While we performed extensive validations and designed our analyses to ameliorate this risk, it is a relevant consideration for all analyses of the presented dataset. Third, we focused our initial analysis of this large dataset primarily on DNA methylation at CpG dinucleotides, given its well-established biological roles. Nevertheless, the RRBS assay also covers DNA methylation at non-CpG sites (i.e., CpA, CpC, CpT), and we observed expectedly low levels of non-CpG methylation in our dataset (species medians ranging from 0.99% to 2.43% across all analyzed vertebrate species). We also detected significantly higher non-CpG methylation levels in brain compared to other tissues in mammals and birds, consistent with a recent report focusing on much fewer species^25^. Fourth, this study relies on our reference-free analysis method (RefFreeDMA)^45^, which enables us to work without reference genomes but lacks the regional context that is provided by a high-quality reference genome. We addressed this limitation by focusing on transcription factor binding sites, whose DNA methylation levels tend to reflect the activity of the corresponding transcription factors. Moreover, we devised a cross-mapping strategy that leverages gene annotations from existing reference genomes and integrates data from different species in a human ortholog gene space. Fifth, despite the large number of samples and species covered by this study, several interesting clades especially among amphibians and reptiles are not well represented in our dataset. Finally, the different species do not provide fully independent data points but are connected through evolution. We thus used statistical methods that correct for phylogenetic relationships, and we evaluated our classifiers both within and across species.

In conclusion, this study provides an initial account of the DNA methylation landscape associated with vertebrate evolution, both by establishing a dataset of unprecedented scale and by deriving insights into conserved and divergent aspects of DNA methylation across a wide range of animal species. Most notably, we found that DNA sequence and DNA methylation exhibit widespread associations in both vertebrate and invertebrate species that gradually changed over the course of vertebrate evolution. The presented data and analyses also provide an evolutionary context for investigating the epigenetic heterogeneity that is observed in human and animal populations and in a broad range of diseases.

## Methods

### Sample collection

The selected samples represent all vertebrate classes and many marine invertebrate species. To obtain this breadth of coverage, samples were obtained from several sources (Supplementary Data 1–4):

1. *Research Institute of Wildlife Ecology of the University of Veterinary Medicine Vienna* (1611 samples): Tissue samples were collected during routine pathological examination of deceased wild, pet, and zoo animals. They were fresh-frozen and stored at −80 °C. Pathological conditions and sample preservation (well preserved, intermediate, rotten) were recorded (Supplementary Data 3). Well-preserved samples were preferentially selected. Species names were obtained from the notes of the pathological examination. Peripheral blood samples of Bactrian camel (*Camelus bactrianus*) and llama (*Lama glama*) were collected as part of routine veterinary examinations. Blood cell types were isolated using forward/side scatter FACS^45^.
2. *Ocean Genome Legacy Center (OGL) at the Northeastern University Marine Science Center* (600 samples): Specimens were collected and deposited to the OGL collections by numerous collaborating researchers and were stored at −80 °C prior to dissection. DNA was isolated using the Qiagen DNeasy Blood & Tissue kit according to the manufacturer’s protocol and stored at −80 °C prior to shipment on dry ice.
3. *Commercial fish farm (Biofisch Wien)* (73 samples): Innards of fish killed for food were immediately dissected, transported on dry ice, and stored at −80 °C until DNA extraction using the Qiagen DNeasy Blood & Tissue kit.
4. *Commercial fish retailer (Naschmarkt Wien)* (67 samples): Whole specimens of sea food were purchased, transported on ice, dissected, and stored at −80 °C until DNA extraction using the Qiagen DNeasy Blood & Tissue kit.
5. *Department of Medical Biochemistry of the Medical University of Vienna* (21 samples): Tissue samples of chicken (*Gallus gallus*) were collected and stored at −80 °C until DNA extraction using the Qiagen DNeasy Blood & Tissue kit.
6. *Max Planck Institute for Evolutionary Biology* (16 samples): Tissue samples of Eurasian blackcaps (*Sylvia atricapilla*) were obtained from birds caught at the Pape Ornithological Station in Latvia (56°9′48′′N, 21°1′35′′E) between end of August and beginning of September 2011, then transported to the University of Ferrara in Italy, where they were held in aviaries until sample collection. Experimental procedures unrelated to this work were carried out during the autumn migratory season 2011, and birds were stored at −80 °C until organs were dissected in 2016 at the MPI for Evolutionary Biology. DNA was isolated using a standard phenol-chloroform extraction protocol and stored in ddH2O at −80 °C.
7. *Department of Biology of the University of Kentucky* (16 samples): Tissue samples of Mexican axolotls (*Ambystoma mexicanum*) were collected and stored at −80 °C until DNA extraction using a standard phenol-chloroform extraction protocol.
8. *Department for Pathobiology of the University of Veterinary Medicine Vienna* (15 samples): Tissue samples of flying snakes (*Chrysopelea*) were collected during routine pathological examination of deceased animals and stored at −20 °C until DNA extraction using the Qiagen DNeasy Blood & Tissue kit.
9. *CeMM Research Center for Molecular Medicine of the Austrian Academy of Sciences* (12 tissue samples): Healthy tissue samples of Tasmanian devils (*Sarcophilus harrisii*) were collected and processed as part of a previously published study that investigated Tasmanian devil transmissible tumors^101^.
10. *St. Anna Children’s Cancer Research Institute* (12 samples): Leukocytes and erythrocytes of zebrafish (*Danio rerio*) were collected from kidneys and blood of adult animals. Cells were dispersed in PBS supplemented with 3% FCS and 2 mM EDTA and sorted by FACS following an established protocol for blood cell populations in zebrafish^102^. Sorted cells were lysed, and DNA was isolated using the Qiagen DNeasy Blood & Tissue kit.

### Taxonomic annotation

All samples were annotated with a scientific (Latin) name and a common (English) name based on the information provided by the sample source. Occasionally, the available information did not support the assignment of the exact species; these samples were assigned genus names rather than individual species names. Moreover, the sequencing data for each species were compared with public reference databases (as described in more detail below), and potential errors or ambiguities were flagged or corrected based on manual inspection. Detailed taxonomic annotations for all included species were obtained from the NCBI database using the *classification* function in the R package *taxize* and manually reviewed for accuracy. In all analyses, marsupials were placed in their own group rather than with the other mammals, given their unique evolutionary history. We used the NCBI Taxonomy Browser (https://www.ncbi.nlm.nih.gov/Taxonomy/CommonTree/wwwcmt.cgi) to create a taxonomic tree for the analyzed species, and we visualized the resulting phylogenetic relationships and associated information using the iTOL software^103^. The resulting annotated species tree is provided for interactive viewing and browsing under the following URL: https://itol.embl.de/tree/841339292169571630660457.

### DNA extraction from tissue samples

DNA from tissue samples was extracted using the Qiagen DNeasy Blood & Tissue kit according to the manufacturer’s protocol. Briefly, small pieces of tissue (around 2 mm^3^) were placed in collection tubes, covered with proteinase-K containing digestion buffer, and shaken overnight at 56 °C. When the tissue samples were completely dissolved, the DNA was bound to spin columns and washed, followed by elution in 50–200 µl nuclease free water, depending on the expected amount of DNA. The DNA concentration was then quantified using a Qubit fluorometer. DNA from blood cells was isolated as previously described^45^, using the Allprep DNA/RNA Mini kit (Qiagen). Between 50,000 and two million cells were lysed in 600 μl Buffer RLT Plus supplemented with 1% β-mercaptoethanol and vortexed thoroughly for at least 5 min. The isolation of DNA and RNA was performed according to the manufacturer’s protocol. DNA was stored at −20 °C.

### DNA methylation profiling by RRBS

Reduced representation bisulfite sequencing (RRBS) was performed as described previously^45,70^, using 100 ng of genomic DNA for most samples, while occasionally going down to 1 ng for samples with low DNA amounts (Supplementary Data 1). To assess bisulfite conversion efficiency, methylated and unmethylated spike-in controls were added at a concentration of 0.1%. For most samples, DNA was digested using the restriction enzymes MspI and TaqI in combination (as opposed to only MspI in the original protocol) in order to increase genome-wide coverage. For certain older samples, only MspI was used (Supplementary Data 1). Restriction enzyme digestion was followed by fragment end repair, A-tailing, and adapter ligation. Finally, the libraries were size selected by performing a 0.75× cleanup with AMPure XP beads (Beckman Coulter, A63881), retaining fragments with lengths of approximately 100 bp to 1000 bp. The amount of effective library was determined by qPCR, and samples were multiplexed in pools of 10 with similar qPCR *C*_*t*_ values. The pools were then subjected to bisulfite conversion, followed by library enrichment with PCR. Enrichment cycles were determined by qPCR and ranged from 6 to 18 (median: 11). After confirming adequate fragment size distributions with Bioanalyzer High Sensitivity DNA chips (Agilent), libraries were sequenced on Illumina HiSeq 3000/4000 machines using the 50 or 60 bp single-read setup.

### Sequencing of unconverted RRBS libraries

To distinguish with confidence between genomic thymines und constitutively unmethylated cytosines (which are read as thymines in bisulfite sequencing), we sequenced one RRBS library for each species without the bisulfite conversion step. Libraries were multiplexed in pools of up to 20 samples, and the pools were subjected to size selection with a 0.6× reverse bead clean up and eluted in 20 µl EB. The amount of effective library in the size-selected pools was determined by qPCR using 1 µl size selected library as input. Based on qPCR *C*_*t*_ values for each pool, the number of PCR enrichment cycles was determined as the *C*_*t*_ minus two, which ranged from 5 to 11 cycles. PCR and qPCR cycler programs were the same as in the RRBS protocol. The enriched libraries were subjected to a 1.0× bead clean up. Library size distributions were assessed on Bioanalyzer High Sensitivity DNA chips (Agilent) and ranged from 260 to 300 bp (mostly 280 bp). Libraries were sequenced on Illumina HiSeq 3000/4000 machines using the 50 or 60 bp single-read setup.

### RRBS data processing

The RRBS data were processed using an updated version of the RefFreeDMA software^45^, which is available on Github (https://github.com/jklughammer/RefFreeDMA). For each species, RefFreeDMA clustered the sequencing data into read stacks corresponding to specific positions in the genome, inferred the genomic DNA sequence as a weighted consensus for each read stack (including both converted and unconverted RRBS libraries), and performed DNA methylation calling for each sample against these consensus reference fragments. Two improvements were introduced in the process of generating the consensus references: (i) Detection and removal of contaminating microbial sequences by mapping all reads to a “decoy” genome consisting of the NCBI BLAST dataset of representative bacterial/archeal genomes and keeping only unmapped reads; (ii) incorporation of unconverted RRBS libraries to enhance detection of consistently unmethylated genomic cytosines. For analyses that focused on the genomic sequence (e.g., k-mer frequencies, sequence-based prediction of DNA methylation), only those consensus reference fragments that were covered by the corresponding uncovered RRBS library were considered, in order to minimize bias. Finally, summary statistics and quality metrics (including mapping rate, number of covered CpGs, conversion efficiency, DNA methylation level, contamination level, pre-fragmentation) were calculated for each sample (Supplementary Data 1 and 4).

### RRBS coverage simulation

To assess the genomic coverage of RRBS across a wide range of species, we simulated the restriction digest and size selection in RRBS for all annotated vertebrate genomes that were available from the UCSC Genome Browser, and we determined the expected RRBS coverage for CpG islands, transcripts, promoters, and repeats in each species. To create in silico RRBS libraries, we first mapped all MspI and TaqI restriction sites in these genomes using the *matchPattern* function in the R package *Biostrings*^104^. The resulting restriction fragments were then filtered to mirror the RRBS size selection step, retaining fragments with a length between 50 bp and 1000 bp. Of these fragments, the first and last 50 bp were registered as simulated RRBS reads. We next identified all CpGs within the genomes using the *matchPattern* function and intersected these coordinates with the regions covered by the in silico RRBS libraries, and with the different types of genomic elements (CpG islands, transcripts, promoters, repeats) using the *findOverlaps* function in the R package *GenomicRanges*^105^. Finally, we calculated the fraction of CpGs within each of the assessed genomic elements that are covered by the in silico RRBS libraries. For each genome, the coordinates of the genomic elements were downloaded from the UCSC Genome Browser website (goldenpath/<genome>/bigZips) using the R package *rtracklayer*^106^. Promoters were defined as the regions 1000 bp upstream to 500 bp downstream of the transcription start sites. The genome sequences were obtained from the corresponding genome assemblies provided by the UCSC Genome Browser.

The following species and genome assemblies were included in the analysis: Vase tunicate (ci3), African clawed frog (xenLae2), armadillo (dasNov3), elephant shark (calMil1), Tibetan frog (nanPar1), green anole (anoCar2), medaka (oryLat2), fugu (fr3), tetraodon (tetNig2), Nile tilapia (oreNil2), kangaroo rat (dipOrd1), stickleback (gasAcu1), Atlantic cod (gadMor1), sloth (choHof1), zebrafish (danRer11), manatee (triMan1), microbat (myoLuc2), mouse (mm39), Garter snake (thaSir1), naked mole-rat (hetGla2), squirrel (speTri2), zebra finch (taeGut2), golden eagle (aquChr2), Chinese hamster (criGri1), Guinea pig (cavPor3), purple sea urchin (strPur2), brown kiwi (aptMan1), mouse lemur (micMur2), Hawaiian monk seal (neoSch1), chicken (galGal6), budgerigar (melUnd1), American alligator (allMis1), African elephant (loxAfr3), Japanese lamprey (petMar3), turkey (melGal5), painted turtle (chrPic1), cow (bosTau9), ferret (musFur1), rabbit (oryCun2), tree shrew (tupBel1), hedgehog (eriEur2), white rhinoceros (cerSim1), wallaby (macEug2), marmoset (calJac4), sheep (oviAri4), megabat (pteVam1), squirrel monkey (saiBol1), cat (felCat9), Tasmanian devil (sarHar1), golden snub-nosed monkey (rhiRox1), pig (susScr11), rhesus macaque (rheMac10), baboon (papAnu4), orangutan (ponAbe3), alpaca (vicPac2), horse (equCab3), green monkey (chlSab2), dog (canFam5), rat (rn7).

Because many reference genomes had an incomplete assembly status and consisted of many scaffold sequences (often exceeding 10,000 scaffolds instead of a few dozen chromosomes), we concatenated individual scaffolds into 20 arbitrary chromosomes, separating the sequences by stretches of 100 Ns. This improved software runtimes and avoided out-of-memory issues. After processing, genomic coordinates based on these artificial chromosomes were ported back to the original coordinate space to match the genome annotations.

### Read coverage analysis

To assess biological and technical effects on our RRBS libraries and on the derived consensus references, each consensus reference fragment was evaluated based on its read coverage across all samples for a given species (Supplementary Fig. 2a). The following classification was applied for each sample: If a fragment had a read coverage of more than half the average coverage in that sample it was considered reliably covered. If a fragment had a coverage of more than four times the average coverage across that sample it was considered highly covered. Next, fragments that were highly covered in >80% of the samples were labeled as “repeat” to indicate that they were likely derived from repetitive genomic regions; fragments that were highly covered in <20% of the samples were labeled as “amplified”, given that this pattern is characteristic of PCR amplification artifacts; and fragments that were reliably covered in >80% of the samples of one individual but in <20% of the samples of other individuals were labeled “private”, as such patterns can arise from inter-individual genetic variability. For each sample, the relative proportion of these three categories (“repeat”, “amplified”, “private”) was calculated and averaged across all samples for a given species. For statistical assessment, only species with at least four samples and at least two individuals were considered.

### Cross-mapping analysis

To validate our consensus references and to enable gene-centric analyses for a subset of species, we devised a cross-mapping workflow that connects the RefFreeDMA-derived consensus reference fragments to putative orthologous regions in available reference genomes. We identified an empirical “best fit” by aligning all consensus reference fragments of a given species to all reference genomes of species in the same class in the UCSC Genome Browser (as determined by the *lowest_common* function in the R package *taxize*). For each species, the reference genome with the highest mapping rate was determined and used for further analysis. Mapping was performed using the cross-mapping function of RefFreeDMA with an allowed mismatch rate of up to 0.2 (this value was empirically determined). The genomes used for cross-mapping and their preparation are described in the *RRBS coverage simulation* section. DNA methylation profiles across annotated genes were created by averaging DNA methylation calls within 5000 bp upstream or downstream of the gene body in bins of 100 bp, and in bins of 200 bp within the gene body itself. For sample-wise analyses the samples were kept separate, whereas all samples of a given species were combined for species-wise analyses.

### Integration of publicly available WGBS data

To validate our RRBS-based, reference-free DNA methylation analysis against WGBS data, we identified the species in our dataset for which WGBS data were publicly available from GEO. This included: *Bos taurus*^57^ (GSE147087), *Mus musculus*^56^ (GSE42836), *Phascolarctos cinereus*^58^ (GSE149600), *Gallus gallus*^66^ (GSE146620), *Parus major*^65^ (SRR2070790), *Xenopus laevis*^62,63^ (GSE76247, GSE90898), *Danio rerio*^60,61^ (GSE149416, GSE134055), *Callorhinchus milii*^25^ (GSE141609), *Branchiostoma lanceolatum*^25,59^ (GSE102144, GSE141609), *Crassostrea gigas*^55^ (GSE40302), and *Octopus bimaculoides*^25^ (GSE141609). In addition, we obtained WGBS data for *Chelydra serpentina* from the authors of the corresponding paper^64^. Supplementary files containing CpG-wise coverage and methylation information were obtained using the R package *GEOquery*^107^ and converted into a common format containing CpG-wise read coverage and DNA methylation ratio. For *Danio rerio* (GSE149416, GSE134055) and *Parus major* (SRR2070790) no suitable files were available; hence we reprocessed the WGBS data for these species starting from the raw sequencing data using the gemBS pipeline^108^ with the danRer11 and the Parus_major1.1 genome assembly, respectively.

### Validation of species annotations

We used our RRBS data to verify species annotations for each sample. First, we created a bisulfite converted version of the NCBI BLAST nucleotide database (Nucleotide collection nr/nt) and mapped 1000 random reads per sample using the NCBI BLAST command line tool with the following parameters: -max_target_seqs 100 -num_threads 4 -word_size 15 -evalue 0.00000001 -outfmt “6 qseqid sseqid sscinames scomnames qlen slen sstart send pident length evalue bitscore qseq” (https://github.com/jklughammer/bisulfiteBlast). Where both of the two best matching species differed by more than the level of “class” from the annotated species (this was assessed using the *lowest_common* function in the R package *taxize*), samples were manually inspected and flagged as unreliable if the discrepancies could not be explained (e.g., by the absence of related species in the NCBI database). In total, 30 samples were flagged as unreliable (Supplementary Data 1).

### Analysis of genome-wide DNA methylation levels

To investigate the association between genome-wide DNA methylation levels and the genomic DNA sequence composition, we calculated mean DNA methylation levels across all CpGs and across all samples for each species, and we correlated these values with three sets of features derived from the corresponding consensus reference: (i) k-mer frequencies; (ii) CG composition; (iii) CpG island frequencies. K-mer frequencies for k ranging from 1 to 3 were calculated using the MEME suite’s *fasta-get-markov* software tool^109^. CG composition included the frequency of C and G nucleotides, the frequency of CpG dinucleotides, the ratio between observed and expected CpG frequencies (where the expected frequency is defined as the calculated combinatory frequency based on independent C and G frequencies), and the absolute number of covered CpG sites. CpG island frequencies were calculated by determining the percentage of consensus reference fragments that fulfilled the Gardiner-Garden or Takai-Jones criteria for CpG islands^110,111^, requiring a GC content (combined C and G frequencies) of at least 50% (Gardiner-Garden) or 55% (Takai-Jones) and a CpG observed versus expected ratio of at least 0.6 (Gardiner-Garden) or 0.65 (Takai-Jones), over stretches of 50 bp.

We evaluated the explanatory power of these feature groups for the observed variation in genome-wide DNA methylation levels across species, using a standard linear model as well as linear models that included the phylogenetic group annotation or the taxonomy tree as additional information. Linear models were implemented in R, using the  package *phylolm*^112^ for the integration of taxonomy tree structure. The variance explained (R^2^) by each of the models was calculated using the *R2.pred* function in the R package *rr2*^113^. The R^2^ values were further adjusted using the Wherry formula to account for the number of variables in each of the models^114^. All models were additionally evaluated by the Akaike information criterion (AIC), using the *AIC* function. To evaluate the predictive power of the 3-mers, we used stepwise feature selection^115^, iteratively adding and removing the features (i.e., individual 3-mers) in the linear model. Models were compared based on the AIC using the *stepAIC* function in the R package *MASS*. Each 3-mer was assigned a stability score calculated as the percentage of bootstrap experiments in which the feature was selected for the final model.

We also assessed how well 3-mer frequencies recapitulate phylogenetic distance between the analyzed species. To that end, we derived a pairwise distance matrix across species based on the global 3-mer frequencies in the consensus reference of each species, using the *dist* function in R package *stats*^116^. We then performed hierarchical clustering of this distance matrix using the *hclust* function from the same package with default parameters, and we visualized the result as a dendrogram using the R package *dendextend*^117^.

Finally, in the analysis of publicly available WGBS data, genome-wide DNA methylation values were calculated for each sample by averaging across the DNA methylation levels of all CpGs with coverage exceeding five reads.

### Generalized linear models controlling for phylogenetic relationships

To test for statistically significant associations between genome-wide DNA methylation levels and 3-mer frequencies (and separately for theoretical cancer risk), we used generalized linear models that explicitly account and control for phylogenetic relationships. Models were built individually for each factor, either with and without taking phylogeny into account, and the corresponding coefficients and associated *p*-values were used for interpretation. The phylogenetic models were built with the *compare.gee* function in the R package *ape*^118^ assuming Gaussian distributions and using the taxonomic tree as depicted in Fig. 1c. The standard models (without controlling for phylogeny) were built using the *glm* function in R. For the 3-mer analysis, the *p*-values obtained from both models were adjusted for multiple testing using the Bonferroni method.

### Analysis of DNA methylation erosion

As a measure of DNA methylation erosion, the proportion of discordant reads (PDR) was calculated as described in the original publication^68^. A custom Python script (which is now part of RefFreeDMA) was used to determine the number of concordantly or discordantly methylated reads with at least four valid CpG measurements for each CpG within each sample. For each CpG, the PDR was then calculated as the ratio of discordant reads compared to all valid reads covering that CpG. CpGs at the end of a read were disregarded as potentially unreliable. Finally, sample-wise PDR values were calculated by averaging across their CpG-wise values.

### Prediction of locus-specific DNA methylation levels

To investigate the association between locus-specific DNA methylation and the underlying DNA sequence, we trained machine learning classifiers to predict the discretized mean DNA methylation levels of individual genomic regions (averaged across samples and/or tissues in a given species) based on their genomic DNA sequence. Specifically, we trained support vector machines (SVMs) with a spectrum kernel from the R package *kebabs*^119^ to predict the discretized DNA methylation states of consensus reference fragments (low: DNA methylation <20% in all samples, high: DNA methylation >80% in all samples; mean coverage >10 reads) based on the DNA sequence composition of the consensus reference fragment. From the set of sequences assigned to high and low DNA methylation state, we randomly selected class-balanced training and test sets comprising 2000 sequences each. In those species where one class contained fewer than 2000 sequences, the number of sequences for the other class was reduced accordingly, in order to avoid class imbalance.

For each species, training and test set sequences were transformed into feature matrices comprising all k-mer frequencies with a fixed length k. Based on the training set, the optimal regularization value (C) as well as the optimal k-mer length (k) were selected by grid search across C values of 0.01, 0.1, 1, and 10, and across k values from 1 to 10, using 10-fold cross-validation (Supplementary Fig. 6b). Finally, SVMs were retrained on the complete training set (without cross-validation) using the optimal parameters and evaluated on the test set. Generation of feature matrices, grid search, and model fitting was done using the R package *kebabs*^119^.

A second set of models was trained and evaluated in each species using only 3-mers. To quantify the predictive power of individual 3-mers in each taxonomic group, we calculated mean feature weights for each 3-mer across all species in that group. These mean feature weights were used to generate sequence logos with the R package *ggseqlogo*^120^, separately for positive and negative feature weights. The significance of differences in the mean feature weights was assessed using the Wilcoxon rank-sum test (*wilcox.test* function in R). For each taxonomic group, the top-10 k-mers with the most significant differential feature weights were reported (Supplementary Fig. 6f). Finally, to test the robustness of these results, all predictions were repeated with less stringent thresholds that included sequences with low DNA methylation levels in any sample as opposed to all samples (low: DNA methylation <20% in any sample, high: DNA methylation >80% in all samples), and the ROC-AUC values were compared (Supplementary Fig. 6a).

For validation by reference-based analysis of WGBS data, we selected those species for which a suitable reference genome and WGBS data with at least two biological replicates were publicly available. The following species and datasets were included: *Bos taurus*^57^ (GSE147087; bosTau9), *Mus musculus*^56^ (GSE42836; mm9), *Gallus gallus*^66^ (GSE146620; galGal5), *Xenopus laevis*^62,63^ (GSE76247, GSE90898; Xla.v91), *Phascolarctos cinereus*^58^ (GSE149600; phaCin_unsw_v4.1), *Danio rerio*^60^ (GSE134055; danRer11), *Branchiostoma lanceolatum*^25,59^ (GSE102144, GSE141609; Bl71nemr), and *Chelydra serpentina*^64^ (data provided by the authors, ASM1885937v1). All genome assemblies were processed using the R package *Biostrings*^104^ and split in 50 bp tiles, mimicking the consensus reference fragments. Each tile was annotated with its mean DNA methylation level calculated as the coverage-weighted mean of DNA methylation values for each CpG in the tile. As in the RRBS-based analysis, only sequences with a mean coverage of at least 10 reads across all samples were retained. Sequences with DNA methylation levels >80% in all samples were labeled “highly methylated”, whereas those with DNA methylation levels <20% in all samples were labeled “lowly methylated”. The support vector machine was trained and tested with cross-validation on a balanced subset of 2000 randomly chosen sequences, while ensuring that test and training sequences did not belong to the same chromosome. This procedure was repeated three times in each species, in order to assess the stability of the results.

### Cross-species predictions and inverted species

To assess the generalizability of locus-specific prediction across species, models were trained in one species and tested (without re-training) in a different species. Model performance in each scenario was quantified by receiver operating characteristic area under curve (ROC-AUC) values in unseen test sets of the target species.

These cross-species predictions unexpectedly resulted in a few cases (13 out of 580 species) in which the observed cross-species prediction performance was systematically lower than expected by chance. We refer to those outliers as inverted species, given that the relationship between locus-specific DNA methylation and the underlying DNA sequence appears to be inverted compared to most other vertebrate and invertebrate species. We denoted species as inverted if they had average ROC-AUC values below 0.45 compared to all other species. The taxonomic group with most inverted species (*actinopteri*) was investigated further. To that end, we identified those 3-mers whose feature weights deviated most strongly in the inverted species, as compared to all other species in this taxonomic group. To test the hypothesis that the observed inversion in the relationship between DNA methylation and DNA sequence may be due to recent expansion of heavily methylated repeats in the inverted species, we used the identified 3-mers for repeat identification, calculating the frequencies of 3-mer derived 9-mer repeats (e.g., ACGACGACG) across all consensus reference fragments with high (>80%) and low (<20%) average DNA methylation levels.

### Analysis of tissue-specific DNA methylation

To assess the prevalence of tissue-specific versus inter-individual differences in DNA methylation, we focused on species with samples for at least two individuals, at least two tissues, and one common tissue that was shared between individuals. We further excluded species that had <50% average CpG overlap between samples or were flagged in the validation of species annotations. For each of the selected species, we calculated the variance explained by the tissue type and by the individual as the average squared Pearson correlation (R^2^) for the mean DNA methylation levels of the consensus reference fragments across samples. The Pearson correlation was calculated using the *cor* function in R. The significance of the difference between the variance explained by tissue and by individual between taxonomic groups was calculated using a two-sided paired Wilcoxon test *(wilcox.test* function in R). Word clouds representing the relative frequency of tissues contributing to the analysis were produced using the *wordcloud* function in the R package *wordcloud*.

Differentially methylated consensus reference fragments between tissues (specifically between heart and liver) were determined by RefFreeDMA as described previously^45^. First, differentially methylated CpGs were identified using the R package *limma*^121^ with multiple-testing correction using the Benjamini–Hochberg method. Second, the *p*-values for individual CpGs within the same consensus reference fragment were combined using a modified version of Fisher’s combined probability test^122^. Third, to identify the top-500 most hypermethylated consensus reference fragments in one tissue compared to the other tissue, we used a combined rank approach based on *p*-value, relative difference, and absolute difference in DNA methylation. Fragments were further required to have a *p-*value below 0.05 and an average coverage of at least two reads in both tissues.

### Transcription factor binding site analysis

To identify enriched transcription factor binding motifs among the differentially methylated consensus reference fragments, we tested the binding position-weight matrixes (PWMs) from the 2020 version of the JASPAR database^123^ using the AME tool from the MEME package with default parameters^124^. We scored each motif for enrichment among the top-500 hypermethylated fragments relative to the top-500 hypomethylated fragments, and vice versa. Motifs with multiple-testing adjusted *p*-values below 0.05 were considered significantly enriched. Transcription factors were additionally annotated based on their gene expression levels in human tissues, using the consensus transcript expression levels from the Human Protein Atlas (https://www.proteinatlas.org/about/download). Only transcription factors that have normalized RNA expression values greater than one in heart or liver samples were included in the analysis. Moreover, to explore the tissue specificity of transcription factor binding, we clustered the corresponding transcription factors based on their motif enrichment in heart and liver using the default hierarchical clustering of the *pheatmap* function in the R package *pheatmap*. GO term annotations of the selected transcription factors were obtained using the GOnet^125^ web tool (https://tools.dice-database.org/GOnet) with a custom set of relevant GO terms (search terms “heart”, “liver”, “hypoxia”, “detoxification”, “fluid shear stress”, “glucagon”, “secretion”, “differentiation”, “regeneration”, “cell cycle”, “glucose homeostasis”, “thyroid hormone”, “nitrogen compound metabolic process”). The resulting network was downloaded as a JSON file and visualized using Cytoscape^126^. For better visualization, connections between GO terms were cut and redundant annotations were removed. Five transcription factors were removed from the network because they were not annotated with any “Biological Processes” GO term (ZNF740, KLF9, ZNF263, ZNF384) or only with the broad term “signal transduction” (KLF16).

Transcription factors were further annotated with their binding preferences (methylated or unmethylated binding site) based on HT-SELEX experiments for their human homologs^86^. Transcription factors annotated as preferring binding to unmethylated sites whose binding sites were hypomethylated in liver (when compared to heart) were classified as “active in liver”. Similarly, transcription factors annotated as preferring binding to methylated sites whose binding sites were hypermethylated in liver (when compared to heart) were also classified as “active in liver”. Transcription factors showing the opposite characteristics were labeled “active in heart”. Using a manually curated database of human gene-regulatory networks^87^, we identified the potential targets of these transcription factors and visualized the resulting network with Cytoscape^126^.

### Reference-based DNA methylation analysis

Using the cross-mapping of RRBS consensus reference fragments to putative orthologous regions in existing reference genomes (as described above) as well as the gene annotations of these reference genomes, we calculated promoter methylation for each covered gene as the mean DNA methylation levels across 50 bins spanning 2500 bp upstream of the transcription start site. Gene identifiers across all genomes were then linked to their human homologs. First, the original RefSeq IDs were converted to NCBI IDs using the NCBI *gene2refseq* dictionary. Second, NCBI IDs were matched to their human orthologs using the NCBI *gene_orthologs* dictionary. Both dictionaries were obtained from https://ftp.ncbi.nlm.nih.gov/gene/DATA.

For an integrated visualization of our dataset, we produced a common dimensionality-reduced representation of genes and samples, combining data across species into a shared human homolog gene space based on mean promoter methylation levels. For the sample-wise analysis, genes were included if they were covered in >100 samples, and samples were included if they had coverage for >400 genes. For the gene-wise analysis, genes were included if they were covered in >400 samples, and samples were included if they had coverage for >50 genes. This filtering strategy was optimized to produce missing-value-free sample-by-sample and gene-by-gene correlation matrices using Person’s correlation with pairwise complete observations as calculated using the *cor* function in R. Uniform Manifold Approximation and Projection (UMAP) was then applied to these correlation matrixes using the *umap* function in the R package *uwot* with the following parameters: n_neighbors = 20, min_dist = 2, spread = 1 (for the sample-wise analysis) or n_neighbors = 15, min_dist = 0.05, spread = 1.5 (for the gene-wise analysis). Leiden clustering for the gene-wise UMAP representation was performed using the *cluster_leiden* function in the R package *igraph* with a resolution of 0.06. Gene enrichment analysis on the genes in each Leiden cluster was performed with the *gost* function in the R package *gprofiler2* using GO Biological Processes, GO Molecular Function, and GO Cellular Compartment as gene set databases and an FDR-corrected *p*-value of 0.05 as the significance threshold.

In the shared human homolog gene space, we assessed the ability to predict sample properties (i.e., tissue type and evolutionary class) based on promoter methylation levels. We used a random forest classifier that can handle missing values, as implemented by the *rpart* function in the R package *rpart*. Genes were included if they were covered in at least 60% of the samples, and samples were included if they had coverage for at least 12% of the genes in the assessed subset of the data. We focused our analysis on mammals and birds as the most highly represented taxonomic groups, and on liver and heart as the most highly represented tissue types. We predicted tissue type (heart versus liver) and evolutionary class (mammal versus bird) separately for mammal and bird species and for heart and liver tissues, respectively. 80 (tissue prediction) and 100 (evolutionary class prediction) species were randomly selected for model training, and the remaining species were used for model testing. We split by species to make sure that training and test data did not contain samples of the same species, thereby focusing the analyses on cross-species prediction. The procedure was repeated 100 times, and for each iteration we recorded the prediction performance (ROC-AUC values) and feature weights. The prediction performance was assessed for each classification task, and average feature weights were calculated across all 100 iterations. As a control analysis, the same procedure was applied to scrambled data derived from the actual data by randomly distributing the non-missing values across the non-missing value positions. This analysis maintained the missing-value structure to ensure that this did not contribute to the observed predictions.

## Supplementary information

Supplementary Information

Editorial Assessment Report

Description of Additional Supplementary Files

Supplementary Data

## Data Availability

All data are available via the Supplementary Website (http://cross-species-methylation.bocklab.org). In addition, the raw and processed data (converted and unconverted RRBS libraries) are available from the NCBI Gene Expression Omnibus (GEO) repository under accession number GSE195869. Previously published datasets used in this study are available from GEO or SRA under the following accession numbers: *Bos taurus* (GSE147087), *Mus musculus* (GSE42836), *Phascolarctos cinereus* (GSE149600), *Gallus gallus* (GSE146620), *Parus major* (SRR2070790), *Xenopus laevis* (GSE76247, GSE90898), *Danio rerio* (GSE149416, GSE134055), *Callorhinchus milii* (GSE141609), *Branchiostoma lanceolatum* (GSE102144, GSE141609), *Crassostrea gigas* (GSE40302), *Octopus bimaculoides* (GSE141609), *Danio rerio* (GSE149416, GSE134055), *Parus major* (SRR2070790).

## Code availability

The source code is available as a ZIP archive on the Supplementary Website (http://cross-species-methylation.bocklab.org), on GitHub (https://github.com/epigen/DNAmeth500species), and as a long-term archive in Zenodo (10.5281/zenodo.7186970).
