# Supplementary material for: Comparative analysis of genome-scale, base-resolution DNA methylation profiles across 580 animal species
Source: Nat Commun. 2023 Jan 16;14:232. doi: 10.1038/s41467-022-34828-y (PMC9842680; doi:10.1038/s41467-022-34828-y)
Supplement: Supplementary file 1 — Supplementary Information [file 41467_2022_34828_MOESM1_ESM.pdf]

SUPPLEMENTARY MATERIAL

Comparative analysis of genome-scale, base-resolution DNA methylation profiles across 580 animal species

Supplementary Figures

Supplementary Figure 1. Summary statistics for 2443 DNA methylation profiles from 580 animal species

Supplementary Figure 2. Read coverage analysis for potential technical and biological sources of variability among the consensus references

Supplementary Figure 3. Cross-mapping of consensus reference fragments to existing reference genomes and analysis of gene-linked DNA methylation patterns

Supplementary Figure 4. Comparative analysis of genome-wide DNA methylation levels across vertebrate and invertebrate species

Supplementary Figure 5. Comparative analysis of DNA methylation erosion and non-CpG methylation across vertebrate and invertebrate species

Supplementary Figure 6. Prediction of locus-specific DNA methylation levels based on the underlying genomic DNA sequence

Supplementary Figure 7. Analysis of “inverted species” with an apparent inversion in the predictiveness of DNA sequence motifs for locus-specific DNA methylation

Supplementary Figure 8. Analysis of variance in locus-specific DNA methylation levels that can be explained by tissues and individuals

Supplementary Figure 9. Analysis of transcription factor binding site (TFBS) motifs among tissue-specific differentially methylated fragments

Supplementary Figure 10. Analysis of DNA methylation at gene promoters across species in the human-ortholog gene space.

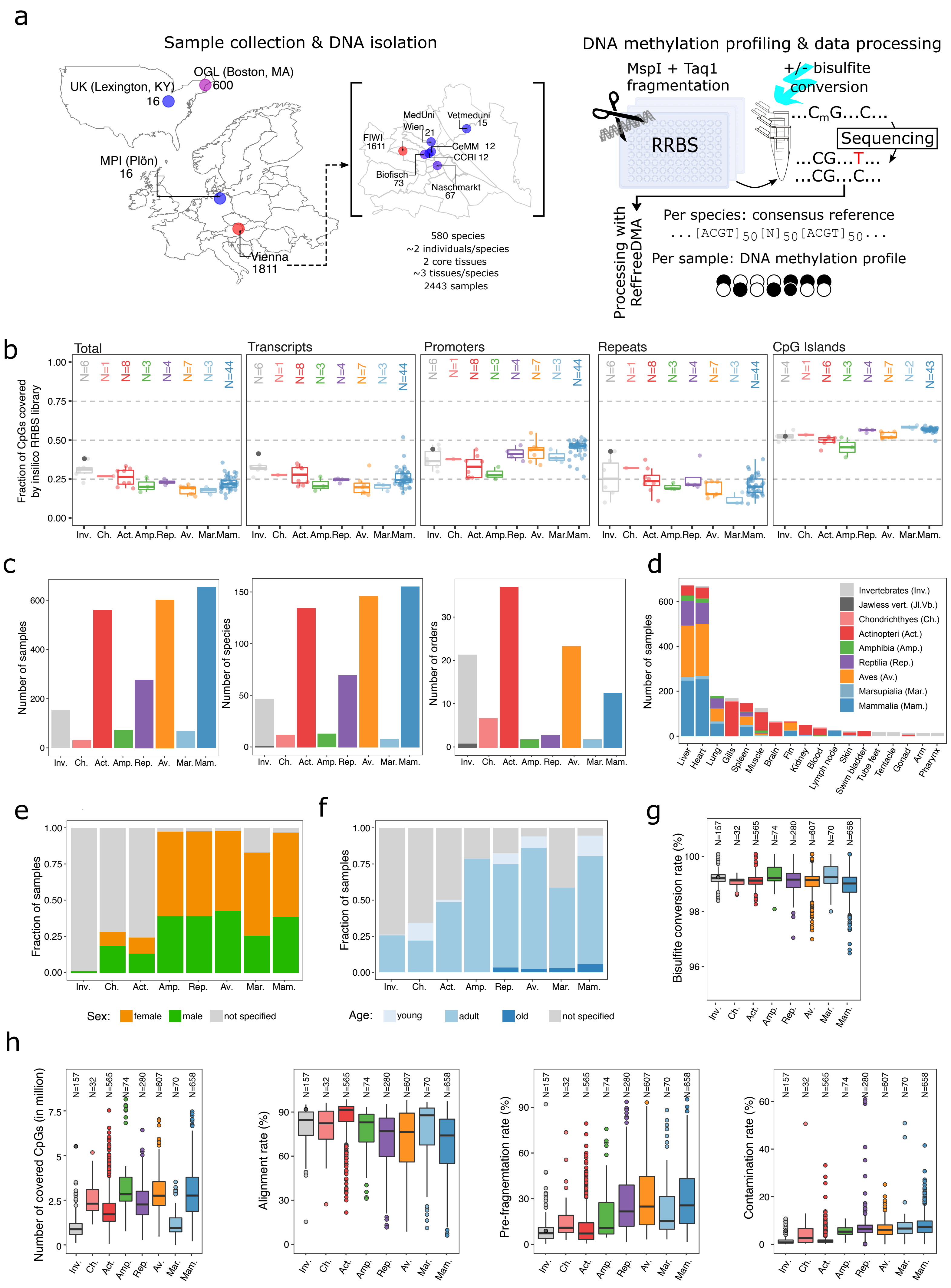

Supplementary Figure 1

Supplementary Figure 1. Summary statistics for 2443 DNA methylation profiles from 580 animal species

(a) Schematic overview of the steps taken to assemble the dataset of vertebrate and invertebrate DNA methylation profiles: Sample collection, DNA isolation, DNA methylation sequencing using the reduced representation bisulfite sequencing (RRBS) assay, and bioinformatic processing using the RefFreeDMA workflow. For each species, an unconverted RRBS library was additionally sequenced to support a more accurate consensus reference reconstruction. Sample sources: FIWI: Research Institute of Wildlife Ecology of the University of Veterinary Medicine Vienna; OGL: Ocean Genome Legacy Center; Biofisch: Commercial fish farm; Naschmarkt: Commercial fish retailer; MedUni: Department of Medical Biochemistry of the Medical University of Vienna; MPI (Plön): Max Planck Institute for Evolutionary Biology; UK: Department of Biology of the University of Kentucky; Vetmeduni: University of Veterinary Medicine Vienna; CeMM: CeMM Research Center for Molecular Medicine of the Austrian Academy of Sciences; CCRI: Children's Cancer Research Institute Vienna.

(b) Boxplots overlayed with dots corresponding to individual species, showing the percentage of CpGs expected to be covered by RRBS in the corresponding species based on simulations throughout the genome (total) and for different types of genomic elements (transcripts, promoters, repeats and CpG islands).

(c) Bar plots showing the number of analyzed samples, species, and orders across all taxonomic groups.

(d) Bar plot showing the representation of different tissue samples across taxonomic groups. Only tissues with more than ten samples are shown.

(e) Stacked bar plot showing the distribution of sex per sample across taxonomic groups.

(f) Stacked bar plot showing the distribution of age per sample across taxonomic groups.

(g) Boxplots showing the bisulfite conversion efficiency per sample across taxonomic groups. For each sample the higher of two measured values (conversion rate at cytosines outside of a genomic CpG context; conversion rate of unmethylated spike-in controls in the RRBS experiment) is displayed.

(h) Boxplots showing RRBS quality control metrics (number of covered CpGs, mapping efficiency, DNA prefragmentation, microbial contamination rate) per sample across taxonomic groups.

Boxplots are specified as follows: center line, median; box limits, upper and lower quartiles; whiskers, 1.5x interquartile range; points, outliers.

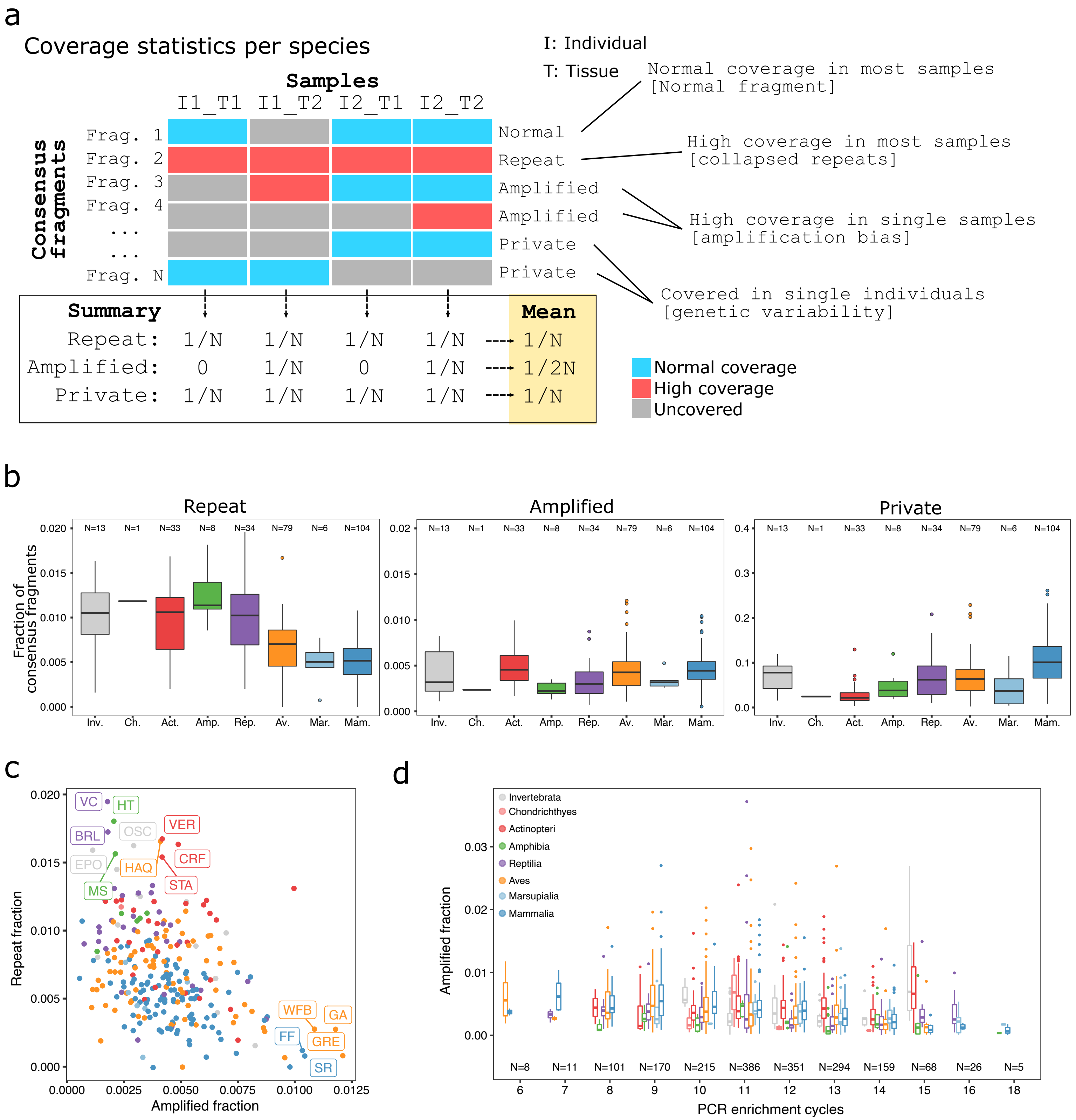

Supplementary Figure 2

Supplementary Figure 2. Read coverage analysis for potential technical and biological sources of variability among the consensus references

(a) Schematic overview for the classification of consensus reference fragments as “repeat”, “amplified”, or “private”, and for the calculation of their frequencies within each species. Consensus reference fragments are classified based on read coverage across all samples in the corresponding species. Sample-wise frequencies of the different classes are calculated and then averaged across all samples to generate species-wise measures.

(b) Boxplot showing the fraction of consensus reference fragments for each of the three coverage classes (“repeat”, “amplified”, “private”) in each of the consensus references as defined in (a), aggregated by taxonomic group.

(c) Scatterplot showing the relationship between the fraction of consensus reference fragments classified as “repeat” and those classified as “amplified” for each of the consensus references, colored by taxonomic group. Species at the extremes are annotated with their abbreviations (**Supplementary Data 2**).

(d) Boxplot showing the fraction of consensus reference fragments classified as “amplified” within each sample, organized by PCR enrichment cycles and aggregated by taxonomic group.

Boxplots are specified as follows: center line, median; box limits, upper and lower quartiles; whiskers, 1.5x interquartile range; points, outliers.

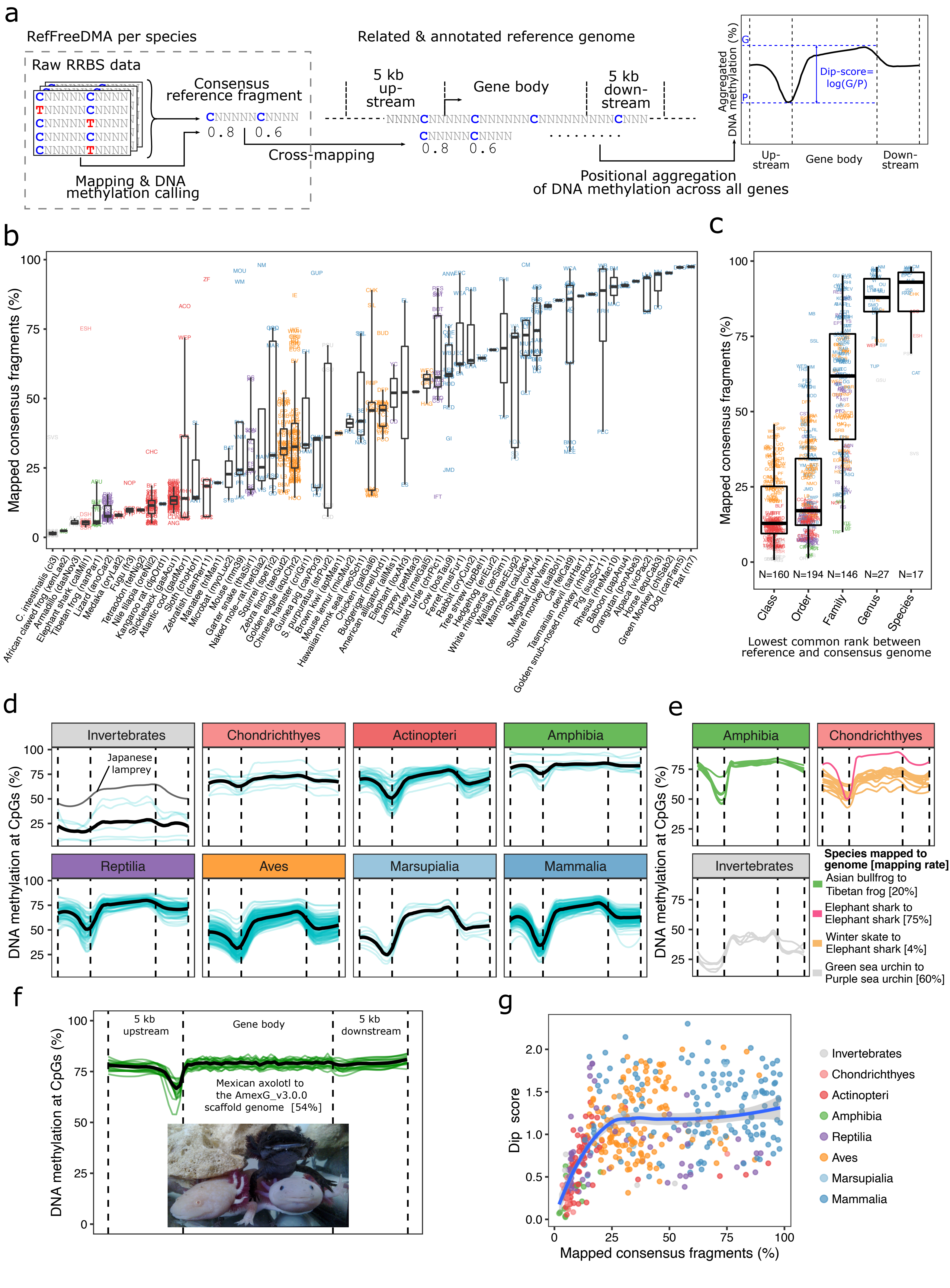

Supplementary Figure 3

Supplementary Figure 3. Cross-mapping of consensus reference fragments to existing reference genomes and analysis of gene-linked DNA methylation patterns

(a) Schematic overview of the cross-mapping approach, which uses gene annotations of related reference genomes to analyze gene-linked DNA methylation patterns including the typical "dip" in promoter regions. This is based on RefFreeDMA-derived consensus reference fragments and their DNA methylation levels, which are cross-mapped to gene-annotated reference genomes. The "dip" in DNA methylation at the promoter region is quantified as the log-ratio of DNA methylation levels at gene bodies (G) and at gene promoters (P).

(b) Boxplot overlayed by data points with the corresponding species abbreviations (**Supplementary Data 2**), showing the mapping rates of all consensus references to their best-matching reference genomes (x-axis), colored by taxonomic group.

(c) Boxplot overlayed by data points with the corresponding species abbreviations (**Supplementary Data 2**), showing mapping rates for all consensus references, aggregated by approximated lowest common rank between consensus reference species and reference genome species, colored by taxonomic group.

(d) Aggregated and smoothed (using loess with span 0.3) DNA methylation profiles across gene annotations including 5000 bp upstream and downstream flanking regions. Each thin line represents one species and thick lines represent the average across all species in the respective taxonomic group.

(e) Same as (d), displaying individual samples for selected species from taxonomic groups with high variation among their aggregated profiles. The species and the reference genome used for mapping are indicated together with the mapping rate.

(f) Aggregated and smoothed (using loess with span 0.03) DNA methylation profiles for the Mexican axolotl, using an earlier scaffold genome assembly because high-quality gene annotations were not available for the most recent chromosome-scale genome assembly of the axolotl.

(g) Scatterplot showing the relationship between the mapping rate of consensus reference fragments and the dip score for species with at least 4000 gene-associated DNA methylation values, colored by taxonomic group. A loess regression curve and a 0.95 confidence interval band fitted to the shown data points are overlaid.

Boxplots are specified as follows: center line, median; box limits, upper and lower quartiles; whiskers, 1.5x interquartile range; points, outliers.

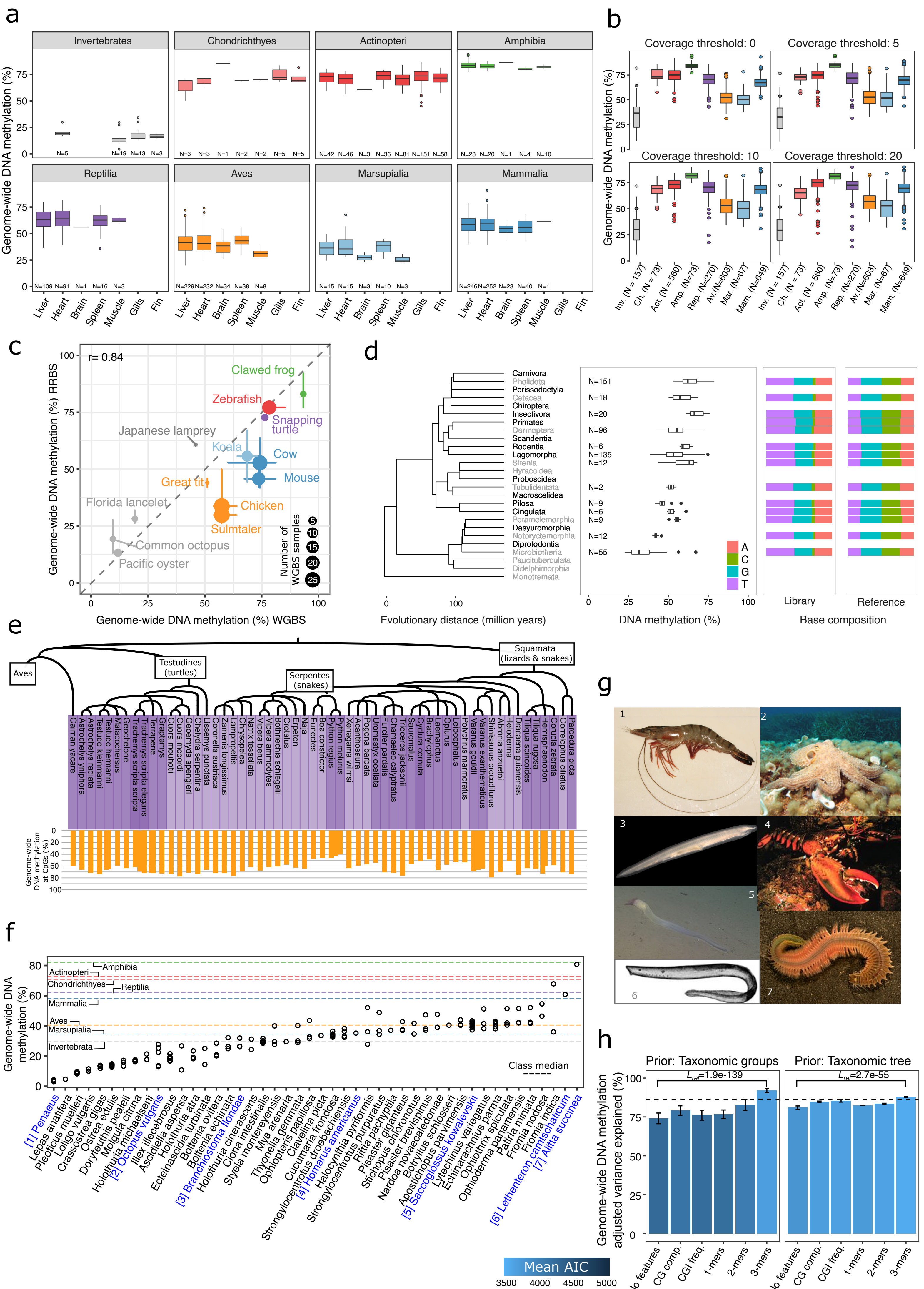

Supplementary Figure 4

Supplementary Figure 4. Comparative analysis of genome-wide DNA methylation levels across vertebrate and invertebrate species

(a) Boxplots showing genome-wide DNA methylation levels per sample, aggregated by taxonomic group and by the seven most abundant tissue types included in this study.

(b) Boxplots showing genome-wide DNA methylation levels per sample for different read coverage thresholds, aggregated by taxonomic group. Only CpGs covered by more than the indicated number of reads were included in this analysis.

(c) Scatterplot comparing genome-wide DNA methylation levels estimated based on RRBS and WGBS data for twelve species. The dot size indicates the number of samples available for WGBS (range: 1 to 42). Error bars depict minimum and maximum sample-wise values in the respective species and assay. The Pearson correlation (r) between the mean genome-wide DNA methylation levels for RRBS and WGBS is indicated.

(d) Boxplot showing mean DNA methylation levels across mammalian orders, including the marsupial orders *diprotodontia* (Australian marsupials, mostly herbivores) and *dasyuromorphia* (Australian carnivorous marsupials). Overlaid are stacked bar plots of base frequencies for the RRBS libraries as well as the consensus reference fragments, indicating broadly similar base frequencies in all mammalian orders. Overlaid on the left is a subset of the taxonomy tree with mammalian and marsupial orders.

(e) Bar plot showing genome-wide DNA methylation levels across reptilian species ordered by their phylogenetic relationships.

(f) Scatterplot showing genome-wide DNA methylation levels for individual samples across invertebrate species as well as the Japanese lamprey (*Lethenteron camtschaticum*). The median of genome-wide DNA methylation levels for all taxonomic groups are indicated as dashed lines for reference.

(g) Images depicting selected species from panel f: Prawn (1), common octopus (2), Florida lancelet (3), American lobster (4), acorn worm (5), arctic lamprey (6), clam worm (7).

(h) Bar plots showing the percentage of variance explained by feature sets reflecting genomic sequence composition (as in Figure 1f), based on linear models that incorporate the taxonomic tree (left) or the taxonomic groups (right) as additional information / priors. Colors indicate the mean Akaike information criterion (AIC), adjusting for model complexity. Error bars represent standard deviations of the mean based on bootstrapping (N=100 iterations).

Boxplots are specified as follows: center line, median; box limits, upper and lower quartiles; whiskers, 1.5x interquartile range; points, outliers.

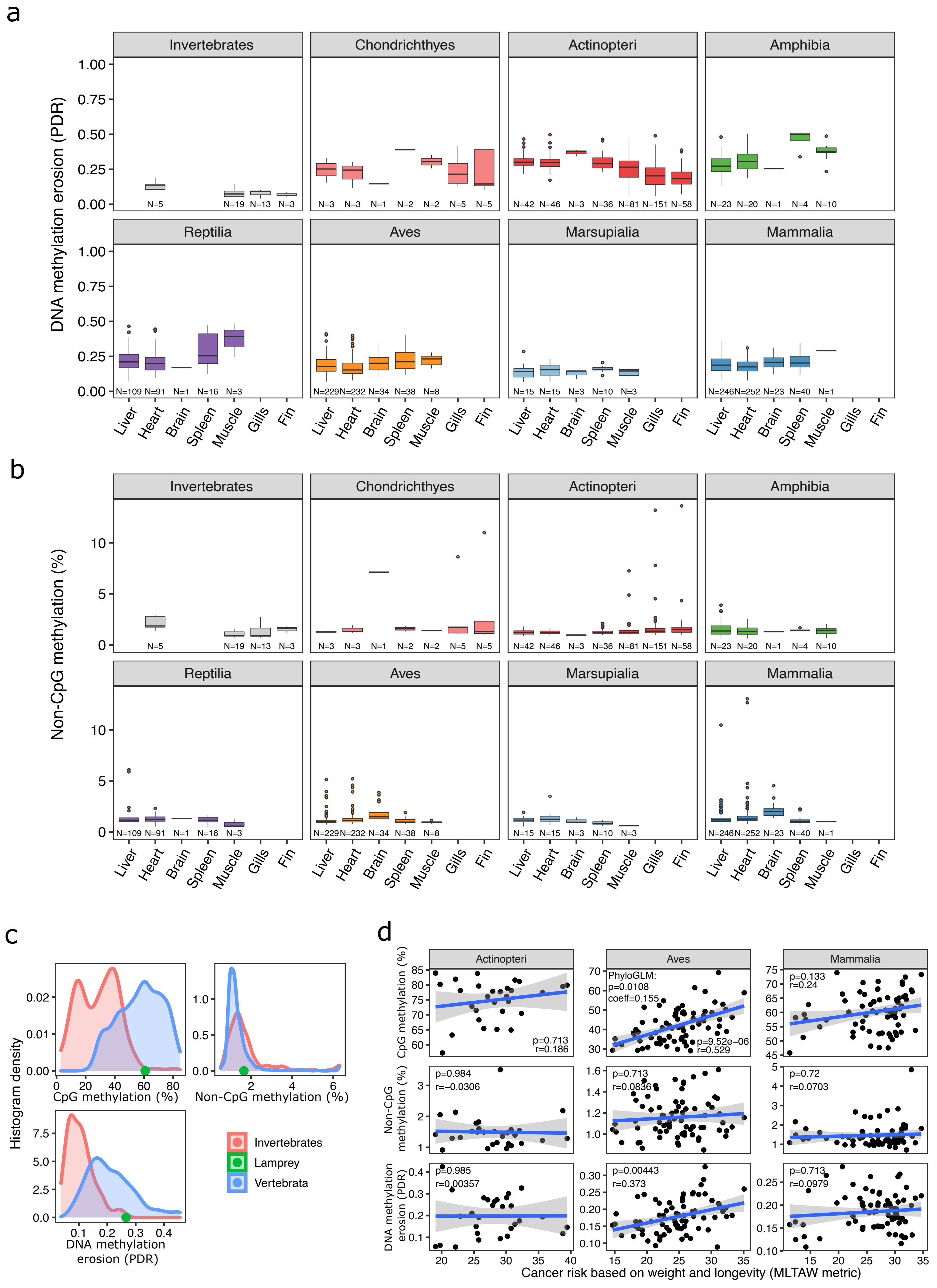

Supplementary Figure 5

Supplementary Figure 5. Comparative analysis of DNA methylation erosion and non-CpG methylation across vertebrate and invertebrate species

(a) Boxplot showing mean proportions of discordant reads (PDR) as a measure of DNA methylation erosion, aggregated by taxonomic groups for the seven most abundant tissue types included in this study.

(b) Boxplot showing mean non-CpG methylation levels, aggregated by taxonomic groups for the seven most abundant tissue types included in this study.

(c) Histograms of genome-wide DNA methylation, non-CpG methylation, and DNA methylation erosion (measured by PDR) for vertebrate and invertebrate species, with the lamprey (an early jawless vertebrate) shown as a green dot.

(d) Scatterplot relating genome-wide DNA methylation levels, non-CpG methylation levels, and DNA methylation erosion (measured by PDR) with theoretical cancer risk estimated by the MLTAW metric, which is calculated using the following empirical formula[79]: MLTAW = log(*Maximum longevity [years]*^6 * *adult weight [g]*). Pearson's correlation coefficient and the corresponding significance (two-sided) are indicated. A linear regression curve with a 0.95 confidence interval is overlaid.

Boxplots are specified as follows: center line, median; box limits, upper and lower quartiles; whiskers, 1.5x interquartile range; points, outliers.

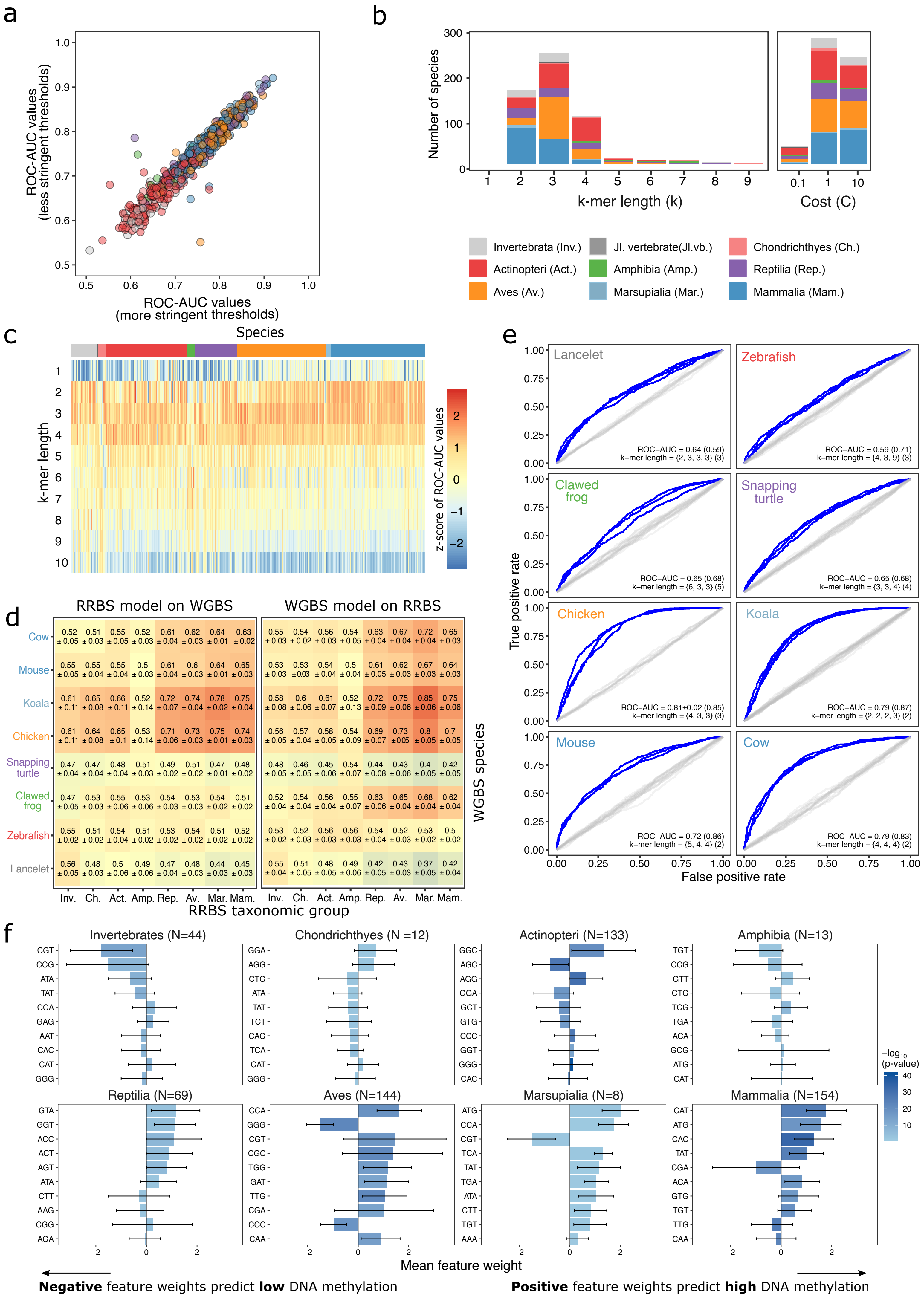

Supplementary Figure 6

Supplementary Figure 6. Prediction of locus-specific DNA methylation levels based on the underlying genomic DNA sequence

(a) Scatterplot comparing the effect of two alternative definitions of fragment-wise DNA methylation states on prediction accuracy measured by ROC-AUC values: Highly methylated fragments (DNA methylation levels above 80% in all samples) and lowly methylated fragments (DNA methylation levels below 20% in any sample, x-axis, or in all samples, y-axis). Each dot corresponds to one species, colored by taxonomic group.

(b) Stacked bar plots displaying the number of species (colored by taxonomic group) for which each k-mer length or learning cost parameter was identified as optimal in a grid search based on the training data.

(c) Heatmap showing scaled ROC-AUC values for a range of k-mer lengths (1 to 10) for prediction of locus-specific DNA methylation across species, colored by taxonomic group.

(d) Heatmap showing the average and standard deviation of the ROC-AUC values for locus-specific DNA methylation prediction when training models based on RRBS data and testing with WGBS data, and vice versa.

(e) ROC curves for prediction of locus-specific DNA methylation based on the 50 bp fragments from underlying genomic DNA sequence using WGBS data for each of the eight indicated species. Three separate (replicate) ROC curves are shown that were obtained based on three non-overlapping sets of sequences (blue). Mean ROC-AUC values as well as favored k-mer lengths for the replicates are indicated in curly brackets, and the corresponding values for RRBS-based DNA methylation data are shown in round brackets. As negative controls, ROC curves trained and evaluated on scrambled data with randomly shuffled labels fall close to the diagonal (in grey).

(f) Bar plots displaying average feature weights for the 10 most predictive 3-mers across taxonomic groups. Error bars denote standard deviations of the mean across all species in the respective taxonomic groups.

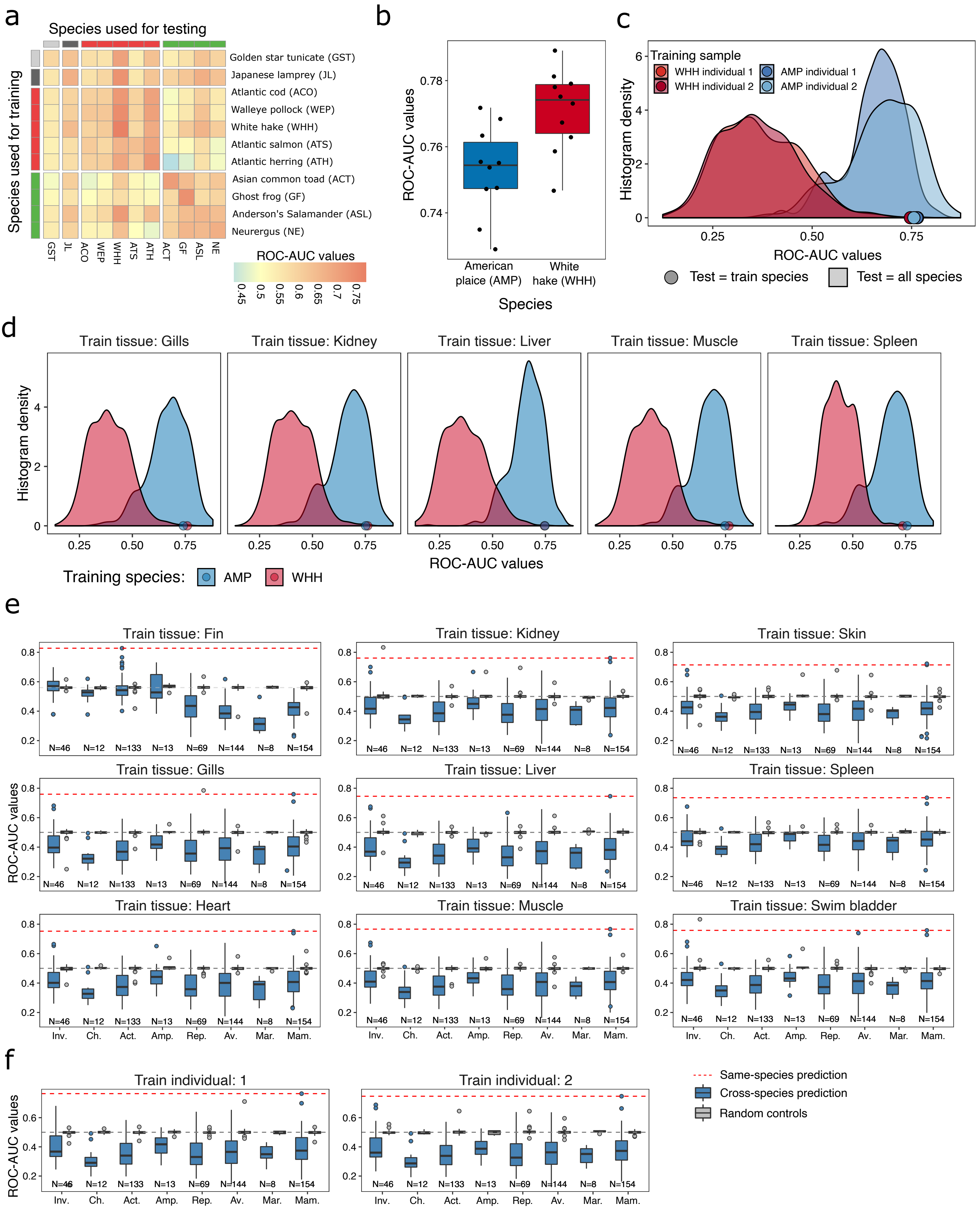

Supplementary Figure 7

Supplementary Figure 7. Analysis of "inverted species" with an apparent inversion in the predictiveness of DNA sequence motifs for locus-specific DNA methylation

(a) Heatmap of cross-species prediction performance (ROC-AUC values), displaying only the inverted species.

(b) Bootstrapping stability (ten different selections of training and test data subsets) for the prediction of locus-specific DNA methylation. The training and testing was performed for white hake (WHH, left), an inverted fish species, and for American plaice (AMP), a non-inverted fish species.

(c) Density plots of cross-species prediction performance (ROC-AUC values) for an inverted species (white hake, WHH, red) in comparison to one representative non-inverted species (American plaice, AMP, blue). Models were trained and tested separately on each individual.

(d) Histogram densities of cross-species prediction performance (ROC-AUC values) for an inverted species and a non-inverted species (as in panel c). Models were trained and tested separately for each tissue type.

(e) Boxplots showing cross-species prediction performance (ROC-AUC values) across all species, using models that were separately trained for the indicated tissues of the inverted species (white hake, WHH). Dashed red lines indicate the ROC-AUC value obtained for the test set from the same species and tissue.

(f) Boxplots showing cross-species prediction performance (ROC-AUC values) across all species, using models that were separately trained for the indicated individuals of the inverted species (white hake, WHH). Dashed red lines indicate the ROC-AUC value obtained for the test set from the same species and individual.

Boxplots are specified as follows: center line, median; box limits, upper and lower quartiles; whiskers, 1.5x interquartile range; points, outliers.

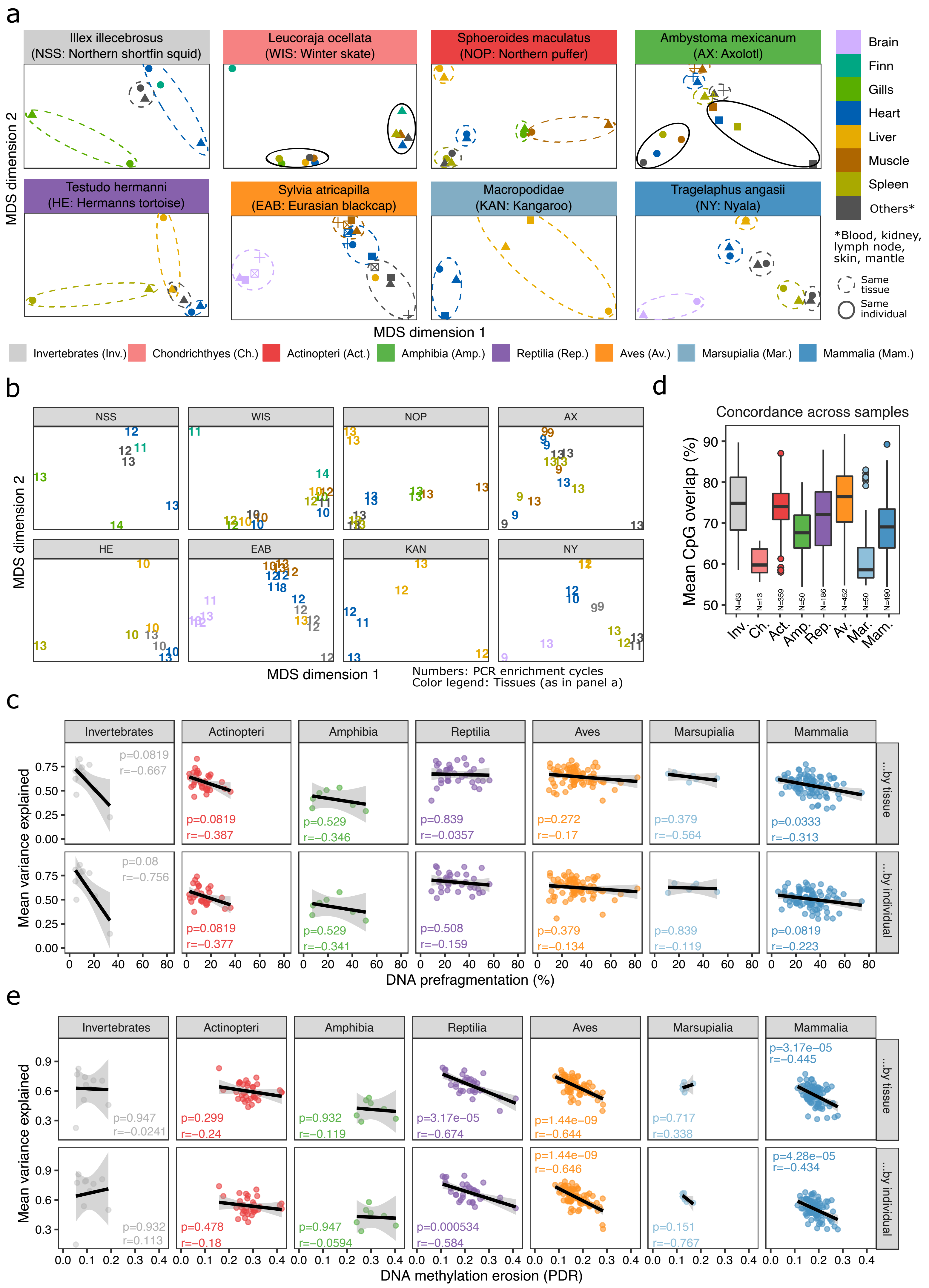

Supplementary Figure 8

Supplementary Figure 8. Analysis of variance in locus-specific DNA methylation levels that can be explained by tissues and individuals

(a) Multidimensional scaling (MDS) plots illustrating the similarity of DNA methylation profiles across tissues and individuals in one selected species for each taxonomic group.

(b) Same as (a) but with individual data points labeled by their PCR enrichment cycles in the RRBS assay.

(c) Scatterplots relating the DNA methylation variance explained by the individual and by the tissue to the amount of DNA pre-fragmentation (as a measure of DNA quality). Each dot corresponds to one species, colored by taxonomic group. Lines indicate linear regressions for each of the taxonomic groups with 0.95 confidence intervals. Pearson's correlation and the associated significance (two-sided) are indicated.

(d) Boxplots displaying the mean overlap in covered CpGs between samples of the same species, relative to the total number of covered CpGs in each sample. This is an indicator of genetic variation in the species, in the sense that more genetically diverse samples tend to have a lower fraction of jointly covered CpGs.

(e) Scatterplots relating the DNA methylation variance explained by the individual and by the tissue to the amount of DNA methylation erosion as measured by the proportion of discordant reads (PDR). Each dot corresponds to one species, colored by taxonomic group. Lines indicate linear regressions for each of the taxonomic groups with 0.95 confidence intervals. Pearson's correlation and the associated significance (two-sided) are indicted.

Boxplots are specified as follows: center line, median; box limits, upper and lower quartiles; whiskers, 1.5x interquartile range; points, outliers.

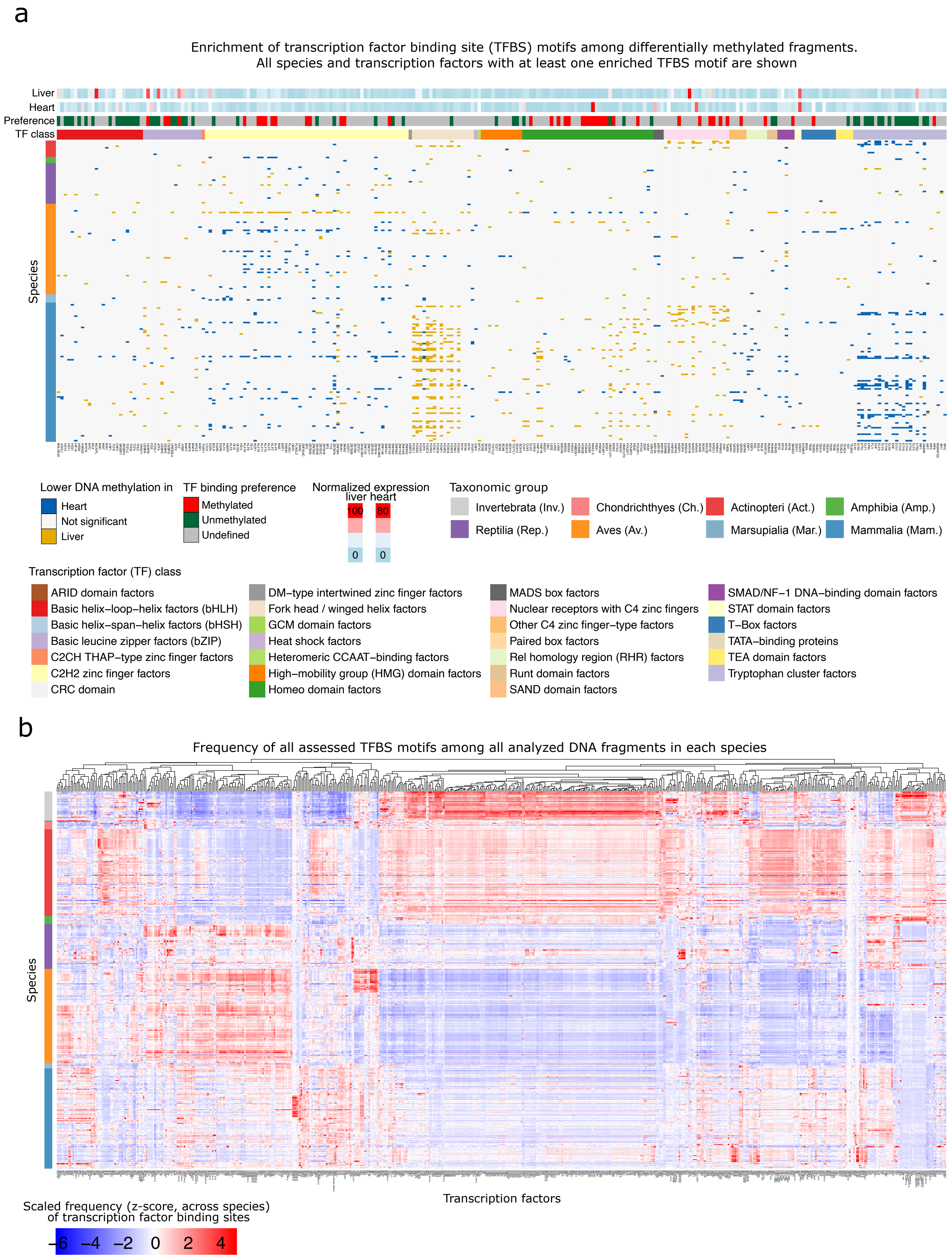

Supplementary Figure 9

Supplementary Figure 9. Analysis of transcription factor binding site (TFBS) motifs among tissue-specific differentially methylated fragments

(a) Sorted heatmap showing TFBS motif enrichments for differentially methylated fragments between heart and liver. For all species (rows) and all transcription factor (columns), the colors indicate whether the corresponding TFBS motifs were enriched in fragments that were hypomethylated in heart (blue) or in liver (yellow). The transcription factors are color-coded by binding preference and transcription factor class (top rows).

(b) Clustered heatmap showing TFBS motif frequencies across all consensus reference fragments for all species (rows) and all transcription factors (columns).

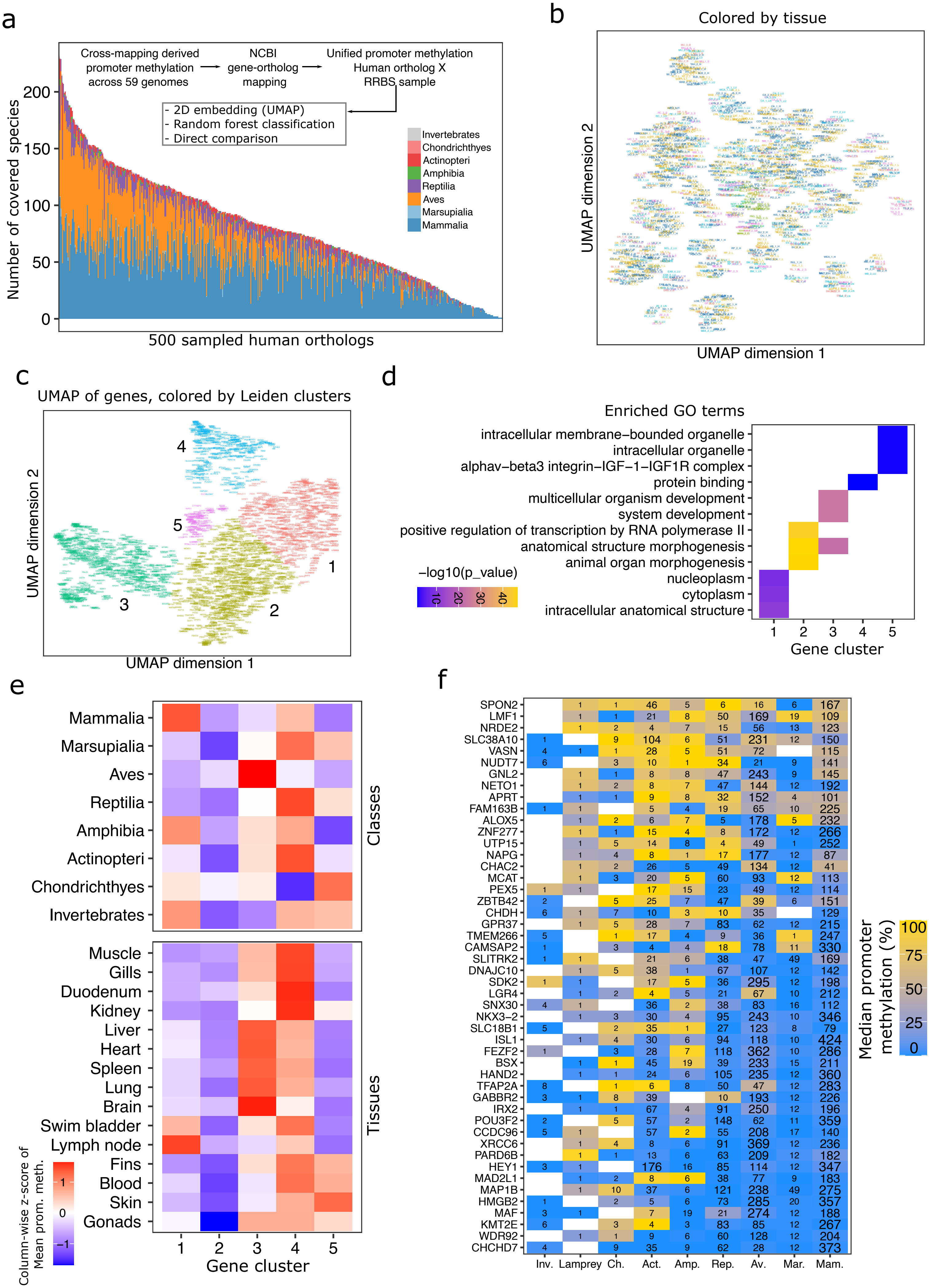

Supplementary Figure 10

Supplementary Figure 10. Analysis of DNA methylation at gene promoters across species in the human-ortholog gene space

(a) Stacked bar plot showing the number of species per taxonomic group for specific human gene orthologs (x-axis). 500 genes were randomly sampled to represent the observed spectrum. Top area: Schematic overview of the mapping of DNA methylation data into the human-ortholog gene space and of the subsequent analysis.

(b) UMAP representation of DNA methylation at gene promoters based on cross-mapping of reference-free consensus reference fragments to annotated reference genomes as in **Figure 5**. Samples are colored by tissue, and each sample is labeled by its searchable sample identifier (**Supplementary Data 1**).

(c) UMAP representation and corresponding Leiden clustering of genes according to their promoter methylation. Genes are colored by Leiden clusters and clusters are numbered. Each gene is labeled by its name, which is searchable and readable when zooming into the PDF of the figure.

(d) Heatmap showing GO term enrichments for the gene clusters defined in panel c. The top three GO terms are displayed for each gene cluster.

(e) Heatmap showing scaled promoter methylation across gene clusters, taxonomic groups, and tissues, filtered at a minimum of eight samples.

(f) Heatmap showing promoter methylation for genes with measurements in most taxonomic groups. Numbers correspond to the number of samples across which median promoter methylation levels were calculated.
