# Supplementary material for: Comparative analysis of genome-scale, base-resolution DNA methylation profiles across 580 animal species
Source: Nat Commun. 2023 Jan 16;14:232. doi: 10.1038/s41467-022-34828-y (PMC9842680; doi:10.1038/s41467-022-34828-y)
Supplement: Supplementary file 2 — Editorial Assessment Report [file 41467_2022_34828_MOESM2_ESM.pdf]

GUIDED OPEN ACCESS

Contents of this report

1. **Manuscript details**: overview of your manuscript and the editorial team.
2. **Review synthesis**: summary of the reviewer reports provided by the editors.
3. **Editorial recommendation**: personalized evaluation and recommendation from all 3 journals.
4. **Annotated reviewer comments**: the referee reports with comments from the editors.
5. **Open research evaluation**: advice for adhering to best reproducibility practices.

About the editorial process

Because you selected the **Nature Portfolio Guided Open Access** option, your manuscript was assessed for suitability in three of our titles publishing high-quality work across the spectrum of genetics research: ***Nature Genetics***, ***Nature Communications***, and ***Communications Biology***. More information about Guided Open Access can be found here.

Collaborative editorial assessment

Your editorial team discussed the manuscript to determine its suitability for the Nature Portfolio Guided OA pilot. Our assessment of your manuscript takes into account several factors, including whether the work meets the **technical standard** of the Nature Portfolio and whether the findings are of **immediate significance** to the readership of at least one of the participating journals in the Nature Portfolio Guided Open Access genetics cluster.

Peer review

Experts were asked to evaluate the following aspects of your manuscript:

- **Novelty** in comparison to prior publications;
- **Likely audience** of researchers in terms of broad fields of study and size;
- **Potential impact** of the study on the immediate or wider research field;
- **Evidence** for the claims and whether additional experiments or analyses could feasibly strengthen the evidence;
- **Methodological detail** and whether the manuscript is reproducible as written;
- Appropriateness of the **literature review**.

Editorial evaluation of reviews

Your editorial team discussed the potential suitability of your manuscript for each of the participating journals. They then discussed the revisions necessary in order for the work to be published, keeping each journal's specific editorial criteria in mind.

Journals in the Nature portfolio will support authors wishing to transfer their reviews and (where reviewers agree) the reviewers' identities to journals outside of Springer Nature.
If you have any questions about review portability, please contact our editorial office at guidedoa@nature.com.

nature portfolio

GUIDED OPEN ACCESS

nature genetics | nature communications | communications biology

Manuscript details

Tracking number	Submission date	Decision date	Peer review type
GUIDEDOA-22-00382	Jan 6, 2022	Feb 23, 2022	Single-blind

Manuscript title

Comparative analysis of DNA methylation across more than 500 animal species

Author details

Christoph Bock

Affiliation: CeMM Research Center for Molecular Medicine of the Austrian Academy of Sciences

Editorial assessment team

**Primary editor**	**Tiago Faial**<br>Home journal: ***Nature Genetics***<br>ORCID: **0000-0003-0864-1200**<br>Email: tiago.faial@us.nature.com
**Other editors consulted**	**Margot Brandt**<br>Home journal: ***Nature Communications***<br>ORCID: **0000-0002-9434-794X**<br>Email: margot.brandt@us.nature.com<br><br>**George Inglis**<br>Home journal: ***Communications Biology***<br>ORCID: **0000-0002-9069-5242**<br>Email: george.inglis@us.nature.com
**About your primary editor**	Tiago Faial obtained his Ph.D. from the Stem Cell and Developmental Biology program at the University of Cambridge under the supervision of Jim Smith and Roger Pedersen, where he studied gene regulatory networks and signaling cascades that underpin mesoderm differentiation. For his postdoctoral work, Tiago joined Joanna Wysocka's laboratory at Stanford University where he studied the dynamics of epigenetic landscapes in pluripotency. He joined the *Nature Genetics* editorial team in 2015 and is based in San Francisco.

Editorial assessment and review synthesis

**Editor's summary and assessment**	The authors generated DNA methylation profiles (RRBS) of 580 animal species (535 vertebrates and 45 invertebrates) using primary tissue/organ samples. Reference-genome-independent analysis of the association between DNA methylation and DNA sequence finds two key transitions: (1) from invertebrates to fish, and (2) from amphibians to reptiles.<br>Cross-species comparisons looking at individual organs support a conserved role of DNA methylation in defining tissue types (more in mammals, birds, and fish; less in reptiles, amphibians, and invertebrates).<br>Cross-mapping analysis of DNA methylation at gene promoters reveals evolutionary changes for certain genes.<br><br>This is an impressive, even if not fully comprehensive or unbiased, survey of DNA methylation patterns in hundreds of species. While there are some novel and potentially interesting findings, the main strength of this work seems to lie in its resource value for evolutionary biologists and the broad community that is interested in DNA methylation dynamics.
**Editorial synthesis of reviewer reports**	Reviewer #1 doesn't note any concerning technical flaws but finds the level of conceptual advance and the resource value limited.<br>Reviewer #2 thinks that this is a fantastic resource. However, they feel that the level of insight is not at the level of *Nature Genetics*, in line with Reviewer #1, but is instead better for *Nature Communications*. The reviewer highlights that the authors could have better integrated phylogenetic data into their analysis.<br>Reviewer #3 is disappointed by the level of insight and thinks that the conclusions about a "DNA methylation code" are poorly supported by the data, mirroring reviewer #1's comment.<br><br>In sum, the three reviewers are underwhelmed by the novelty and/or the degree of conceptual advance provided by the findings, at least for consideration at *Nature Genetics*, but Reviewers #2 and #3 think that this is a valuable dataset that merits rapid publication.

Editorial recommendation

Nature Genetics<br><br>Revision not invited	While the reviewers overall appreciate the value and scale of the effort, they feel that the level of conceptual advance/novelty does not warrant further consideration at *Nature Genetics*.
Nature Communications<br><br>Major revisions	*Nature Communications* would be interested in a revised manuscript that incorporates all of the specific suggestions from reviewers including toning down claims and discussing limitations, as well as the phylogenetic analyses suggested by Reviewer #2.
Communications Biology<br><br>Major revisions	*Communications Biology* would be interested in a revised manuscript that incorporates the phylogenetic analyses suggested by Reviewer #2, while also carefully discussing limitations and qualifying the conclusions, as outlined by all three reviewers.

Next steps

**Editorial recommendation 1:**	Our top recommendation is to revise and resubmit your manuscript to *Nature Communications.* We feel the additional analyses required are reasonable.
**Editorial recommendation 2:**	You may also choose to revise and resubmit your manuscript to *Communications Biology.* This option might be best if the requested revisions are not possible/feasible at this time.
**Note**	As stated on the previous page, *Nature Genetics* is not inviting a revision at this time. Please keep in mind that the journal will not be able to consider any appeals of their decision through Guided Open Access.

Revision

To follow our recommendation, please upload the revised manuscript files using **the link provided in the decision letter**. Should you need assistance with our manuscript tracking system, please contact Adam Lipkin, our Nature Portfolio Guided OA support specialist, at guidedOA@nature.com.

Revision checklist

- ☐ Cover letter, stating to which journal you are submitting
- ☐ Revised manuscript
- ☐ Point-by-point response to reviews
- ☐ Updated Reporting Summary and Editorial Policy Checklist
- ☐ Supplementary materials (if applicable)

Submission elsewhere

If you choose not to follow our recommendations, you can still take the reviewer reports with you.

Option 1: Transfer to another Nature Portfolio journal
Springer Nature provides authors with the ability to transfer a manuscript within the Nature Portfolio, without the author having to upload the manuscript data again. To use this service, **please follow the transfer link provided in the decision letter.** If no link was provided, please contact guidedOA@nature.com.

> *Note that any decision to opt in to In Review at the original journal is not sent to the receiving journal on transfer. You can opt in to In Review at receiving journals that support this service by choosing to modify your manuscript on transfer.*

Option 2: Portable Peer Review option for submission to a journal outside of Nature Portfolio
If you choose to submit your revised manuscript to a journal at another publisher, we can share the reviews with another journal outside of the Nature Portfolio if requested. You will need to request that the receiving journal office contacts us at guidedOA@nature.com. We have included editorial guidance below in the reviewer reports and open research evaluation to aid in revising the manuscript for publication elsewhere.

Annotated reviewer reports

The editors have included some additional comments on specific points raised by the reviewers below, to clarify requirements for publication in the recommended journal(s). However, please note that all points should be addressed in a revision, even if an editor has not specifically commented on them.

Reviewer #1 information

**Expertise**	DNA methylation; evolution

Reviewer #1 comments

Section	Annotated Reviewer Comments
**Remarks to the Author:**	In this manuscript, Klughammer et al. describe the results of analyzing DNA methylation patterns in 535 vertebrate and 45 invertebrate species. While the study is based on a large amount of data (generated by reference-independent RRBS), the findings are very vague. I also found the paper to be poorly accessible and it was very difficult to define its potential impact and scientific value. The authors summarize this as contributing “an epigenetic perspective to the investigation of vertebrate evolution” and providing a “major resource for dissecting the role of DNA methylation in vertebrates and invertebrates” (cited from the last paragraph of the introduction). As explained in my comments below, I found the “epigenetic perspective” very trivial and the value of the “resource” very much limited by technical aspects.<br>**Please carefully proofread the manuscript for clarity, to improve readability and accessibility.**<br><br>1. The authors mention a “genetic code” that underpins DNA methylation patterns. While the unambiguous definition of such a code could be of great value for the epigenetics community, the paper does not provide this. Nor does it “crack” the code in the sense that it provides a tool for accurately predicting DNA methylation patterns.<br><br>2. The paper does not leverage its findings into a conceptual advance. How can this study advance our understanding of vertebrate evolution? What are the evolutionary forces that drive the described changes in DNA methylation patterns at the vertebrate base and then during the emergence of reptiles?<br>**The limited conceptual advance and sample size (per point #3 below) prohibits further consideration by *Nature Genetics*.**<br><br>3. The groundbreaking potential of the paper is greatly limited by its random sampling strategy, low number of biological replicates and its strong focus on vertebrates. A better coverage of the much more diverse invertebrate methylomes

	could have provided substantially more evolutionary insight. For example, higher numbers of replicates per species would be required to substantiate conclusions regarding inter-tissue variation and inter-individual variation.<br>**The sampling strategy (including the focus on RRBS, per point #4 below) should be discussed as a limitation for further consideration by *Nature Communications* or *Communications Biology*.**<br><br>4. The value of the dataset as a resource is greatly limited by its focus on reference-free RRBS. RRBS covers only a limited part of the genome (usually a few percent) and comparability of RRBS data between species is limited by differences in genome structures (CpG density, abundance of CpG islands, etc.). Furthermore, high-quality reference genomes will not be available for the vast majority of species for the foreseeable future, which precludes more detailed downstream analyses.

Reviewer #2 information

**Expertise**	DNA methylation; evolution

Reviewer #2 comments

Section	Annotated Reviewer Comments
**Remarks to the Author: Overall significance**	The authors present a fantastic resource comparing DNA methylation across species. The technical bases of the results that they present, in particular the inference of methylation levels in the different species, seems sound. The resource will be extremely useful to the community and thus is worthy of publication. I note further that the authors have already responded to a set of queries that were raised by a previous round of reviews, and these responses seem valid. I am in favour of rapid publication in order to release the data for the community and in order to spare the authors further rounds of revisions.<br><br>Importantly however the scope of the inferences that the authors actually make from the data is limited and I think that more could be made of the data, potentially in follow-up studies or by others once the resource is released. In particular, the limited way in which the authors attempt to correct for phylogenetic relationships between species in the trends that they identify reduces the weight of their results. I think that in some cases, improving these inferences would be a considerable body of work and could form the basis of a new study. I would therefore advocate that the report should be published and the text of the manuscript appropriately edited to take into account this limitation.<br>**Please elaborate on limitations and potential future directions, for**

	**further consideration at *Nature Communications* or *Communications Biology*.**
**Remarks to the Author: Impact**	The dataset and the possibilities that come along with that are the most important aspect of this manuscript.
**Remarks to the Author: Strength of the claims**	Major concerns<br><br>1) “To assess the relationship between DNA methylation and genome composition across species, we constructed linear models based on a range of features that globally describe the species’ genomic DNA sequence (e.g., k-mer frequencies, CG composition, CpG island frequency). Strikingly, 3-mer frequencies explained more than 80% of the observed variance in mean DNA methylation levels across vertebrate evolution”<br><br>This analysis is confounded by the fact that the phylogeny of the species has not been explicitly taken into account within the linear model. As a result any trends here could be driven by phylogeny rather than altered sequence composition. The authors have tried to discount this by comparing the result to the result when phylogeny is included, and finding that it is better. However, I don’t think that this is the best way to do the analysis. Instead, the authors should construct a phylogenetic glm with a phylogenetic tree as an input as well as the DNA methylation levels and the genomic DNA content. Then, any factors that correlate with DNA methylation independently of the phylogeny would be identified as significant. Otherwise the unequal sampling of the phylogeny, combined with the fact that DNA methylation tends itself to covary with phylogenetic relationships, makes the conclusion weak.<br>**This point (along with points #1-2, as below) would be necessary for further consideration at *Nature Communications or Communications Biology*.**<br><br>2) “We thus investigated the relationship between our global metrics of DNA methylation and estimates of theoretical, unmitigated cancer risk based on each species’ body weight and longevity”<br><br>Exactly the same critique as in 1) applies here. The correlation observed could be driven by phylogeny rather than DNA methylation levels and so the authors would need to take this into account to do the analysis properly, using a phylogenetic model.<br><br>3) In aggregate, our results support the existence of a “genomic code” that links locus-specificDNA methylation levels to the underlying DNA sequence in vertebrate and invertebrate species<br><br>Again, same critique as above. They need to take into account phylogeny otherwise these “codes” could simply be because sequence covaries with

	phylogeny, which, separately, covaries with DNA methylation. It is not adequate that they have considered large taxonomic groups separately because within e.g. mammals there is still unequal sampling across the inter-species differences. In this case correcting this analysis seems complex- it would require an entirely new approach to the machine learning. My approach here would be to take the DNA methylation level and attempt to explain it by the phylogenetic relationship and then use the residuals from this fit as the input for the classifier, but other approaches that take both phylogeny and sequence in one go could be possible too. If this is too involved to implicate, the authors need again to caveat their results accordingly by pointing out that these differences cannot be shown formally to covary with DNA methylation.

Reviewer #3 information

**Expertise**	DNA methylation

Reviewer #3 comments

Section	Annotated Reviewer Comments
**Remarks to the Author**	Bock and colleagues provide an impressive collection of RRBS DNA methylation datasets from 580 animal species.<br><br>It is well known that DNA methylation patterns vary between species in that vertebrates have highly methylated genomes at CGs with the exception of promoters and enhancers including CpG islands leading to a consensus model that DNA methylation is the default state that regulatory regions are protected from a high CG density in case of CpG islands and by dynamic changes in methylation as a function of demethylation in case of CG poor elements. Invertebrates have sparsely methylated genomes since here methylation is targeted to selective sites to mostly repeats and actively transcribed genes. Notable variations to this theme have been studied before (reviewed in Mendoza et al., 2019, Suzuki and Bird, 2008, both overview articles on the topic that warrant citation).<br><br>Exploring these variations further by including additional species is in principle important in order to be able to generalize or spot differences with potential functions.<br><br>The current manuscript illustrates the potential of RRBS to enable the study of many samples as only a subfraction of the genome but also its limitations in that this subfraction is also dependent on restriction site occurrences, which

	are dependent on CG content and DNA methylation, which is not possible to fully account for in the absence of reference genomes and thus somewhat limits the use as a reference.<br><br>Key reported findings and concerns:<br><br>- similarity in DNA methylation levels are high between related species but that these vary rather widely overall suggesting differences in DNA methylation maintenance. For this reviewer the most interesting observation.<br><br>- Non-CG methylation appears limited to brain tissue from mammals and birds, which had been previously only observed before in mammals.<br><br>- tissue-specific differences in DNA methylation are linked to transcription factor activity. This is not novel but has been extensively reported before in species with genome wide methylation (labs of Lister, Ecker, Schubeler and others) including a recent example in a sponge (Mendoza, Nature Eco & Evo 2019).<br><br>- the authors further argue that they discovered a genomic code for DNA methylation due to differences in trinucleotides that explain DNA methylation pattern. This is a strong claim as it implies to have decoded how sequences are targeted (rather than finding statistically significant differences in trinucleotide abundances). Indeed this claim seems insufficiently supported by the data as it cannot be excluded that this reflects sequence variations between species that reside in those regions that are methylated including different repeats and overall nucleotide composition. The authors compare this to the nucleosome position code as reported by Segal et al.. It is important to note that the conclusions of this paper have meanwhile been challenged by several groups and are now considered by the community to reflect a flawed statistical analysis and a signal of almost no predictive power in explaining in vivo patterns of nucleosomes. (https://genome.cshlp.org/content/17/8/1170.long, https://pubmed.ncbi.nlm.nih.gov/23463311/, https://pubpeer.com/publications/34904859EA5787B3927F952E0EED43#null). This obviously does not exclude that there is a "DNA methylation code" but given that we know already about molecular preferences of DNMT3 to certain chromatin marks, how can one exclude that these differences are only reflecting differences in sequences of targets such as regulatory regions, repeats and transcribed genes? Are the authors proposing that DNMT interaction with short DNA sequences directly account for these differences? This reviewer advises strongly against the use of the term "code" in this context as it implies information of high predictive power rather than a statistically significant difference with limited predictive power.<br>**Please qualify this result, for further consideration at *Nature Communications* or *Communications Biology*.**

The authors report some remarkable exceptions such as the white hake, which seems only superficially analyzed. It remains unclear if global patterns are shifted at the level of the epigenome or at the level of the genome, a more thorough analysis might lead to more relevant and thought-provoking insights and the evolution of DNA methylation.

The data interpretation somewhat ignores known fundamental differences in genome-wide versus targeted DNA methylation and dinucleotide composition, which seems to lead to oversimplifications.

In summary, this is an impressive large dataset of DNA methylation that should be more cautiously interpreted. In light of the amount of work, the actual novel observations remain somewhat limited and the postulated key observation appears misleadingly overstated. At the same time, the work has obvious merit as a resource, the potential of which seems underdeveloped.

Other points:
Some of the speculations seem overly creative. E.g. to suggest that difference in promoter methylation of one gene could account for lower cancer incidences in birds versus mammals is rather wild.

> **Please tone down some of the more speculative conclusions, such as this example, for consideration at *Nature Communications*.**

It would be helpful to provide additional information of the studied genomes (such as genome-size, repeat abundance, nucleotide frequency, and CpG O/E ratio), where there is a reference genome available. This would help to put the genome-wide methylation levels determined in this study into context of the genomic makeup.

Figure 5c: Locus-specific is misspelled in the y-axis and yellow as a color choice to represent liver methylation is poorly visible and difficult to read.

Transcription factor column names are not readable in Supplementary Figure 9b in my printed version of the manuscript. It might be helpful to remove this label and only highlight individual TFs.

Similarly, data “points” in Supplementary Figure 10b-c are not readable. The authors might consider normal circles or dots.

Open research evaluation

Guidelines for Transparency and Openness Promotion (TOP) in Journal Policies and Practices ("TOP Guidelines")

The recommendations and requests in the table below are aimed at bringing your manuscript in line with common community standards as exemplified by the TOP Guidelines. While every publisher and journal will implement these guidelines differently, the recommendations below are all consistent with the policies at Nature Portfolio. In most cases, these will align with TOP Guidelines Level 2.

FAIR Principles

The goal of the recommendations in the table below related to **data or code** availability is to promote the FAIR Guiding Principles for scientific data management and stewardship (*Scientific Data* **3**: 160018, 2016). The FAIR Principles are a set of guidelines for improving 4 important aspects of digital research objects: **F**indability, **A**ccessibility, **I**nteroperability and **R**eusability.

ORCID

ORCID is a non-profit organization that provides researchers with a unique digital identifier. These identifiers can be used by editors, funding agencies, publishers, and institutions to reliably identify individuals in the same way that ISBNs and DOIs identify books and articles. Thus the risk of confusing your identity with another researcher with the same name is eliminated. **The ORCID website** provides researchers with a page where your comprehensive research activity can be stored.

Springer Nature collaborates with the ORCID organization to ensure that your research contributions (as authors and peer reviewers) are correctly attributed to you. Learn more at https://www.springernature.com/gp/researchers/orcid

Data availability
**Data Availability Statement**
Thank you for including a Data Availability statement. While you have included some important information, the editors have noted that some details appear to be missing. The Data Availability Statement should be as detailed as possible and include accession codes or other unique IDs for deposited data, information about where source data can be found, and specify any restrictions to data access that may apply. At a minimum, the statement should indicate that data are available upon request and explain how data access can be granted. If data access is not possible, the reasons for this must be made clear in the Data Availability Statement.<br><br>More information about the Nature Portfolio data availability policy can be found here: https://www.nature.com/nature-portfolio/editorial-policies/reporting-standards#availability-of-data<br><br>More information about formatting Data Availability Statements can be found here: http://www.springernature.com/gp/authors/research-data-policy/data-availability-statements/12330880
**Mandatory data deposition**
Most scientific journals, including all Nature Portfolio journals, require that any newly-generated DNA sequence data must be made publicly available before publication. There are some exceptions allowed for sensitive clinical data, but this should be discussed with the editor. All data must be deposited in a community-approved repository and accession codes/unique IDs must be included within the Data Availability Statement in the manuscript.<br><br>Examples of appropriate public repositories are listed below:<br><br>-GenBank<br>-Sequence Read Archive (WGS or WES data)<br>-The European Nucleotide Archive (ENA)<br><br>More information on mandatory data deposition policies at the Nature Portfolio can be found at http://www.nature.com/authors/policies/availability.html#data<br><br>Please visit https://www.springernature.com/gp/authors/research-data-policy/repositories/12327124 for

a list of approved repositories for various data types.
**Other data requests**
In line with community standards regarding open research, Springer Nature strongly supports data sharing and believes that all datasets on which the conclusions of the paper rely should be available to readers. We encourage authors to ensure that their datasets are either deposited in publicly available repositories (where available and appropriate) or presented in the main manuscript or additional supporting files whenever possible.<br><br>To learn more about data sharing and recommended data repositories, please see https://www.springernature.com/gp/authors/research-data-policy/repositories/12327124
**Data citation**
Please cite (within the main reference list) any datasets stored in external repositories that are mentioned within their manuscript. For previously published datasets, we ask that you cite both the related research article(s) and the datasets themselves. For more information on how to cite datasets in submitted manuscripts, please see our data availability statements and data citations policy:<br>https://www.nature.com/documents/nr-data-availability-statements-data-citations.pdf<br><br>Citing and referencing data in publications supports reproducible research, by increasing the transparency and provenance tracking of data generated or analyzed during research. Citing data formally in reference lists also helps facilitate the tracking of data reuse and may help assign credit for individuals' contributions to research. A number of Springer Nature imprints are signatories of the Joint Declaration on Data Citation Principles, which stress the importance of data resources in scientific communication.
**Code availability and citation**
Thank you for making your custom code available via Github. Upon publication, Nature Portfolio journals consider it best practice to release custom computer code in a way that allows readers to repeat the published results. Code should be deposited in a DOI-minting repository such as Zenodo, Gigantum or Code Ocean and cited in the reference list following the guidelines described in our policy pages (see link below). Authors are encouraged to manage subsequent code versions and to use a license approved by the open source initiative.<br><br>See here for more information about our code availability policies:<br>https://www.nature.com/nature-portfolio/editorial-policies/reporting-standards#availability-of-computer-code

Ethics
We believe that authors, peer reviewers and editors should be required to disclose any competing interests that might influence their decisions and conclusions around a particular piece of content. In the interests of transparency and to help readers form their own judgements of potential bias, Nature Portfolio journals require authors to declare any competing financial and/or non-financial interests in relation to the work described.<br><br>Please provide a 'Competing interests' statement using one of the following standard sentences:<br><br>1. The authors declare the following competing interests: [specify competing interests]<br>2. The authors declare no competing interests.<br><br>See the Nature Portfolio competing interests policy for further information:<br>https://www.nature.com/nature-research/editorial-policies/competing-interests<br>The Springer Nature policy can be found here:<br>https://www.springernature.com/gp/policies/editorial-policies
We believe that Springer Nature has a responsibility to support the relevant guidelines (based on research community or geographical region) that specify best practice in research and thus require all experimental results on animal and human participants to conform to the authors' local regulations and ethical standards, and we also encourage adherence to international standards.<br><br>Because your study uses live vertebrates, a statement affirming that you have complied with all relevant ethical regulations for animal testing and research is necessary. A statement explicitly confirming if the study received ethical approval, including the name of the board and institution that approved the study protocol is also required. The species, strain, sex and age of animals should be included.<br><br>Further details on our policies can be found at<br>https://www.nature.com/commsbio/editorial-policies/ethics-and-biosecurity
**Reporting & reproducibility**

We believe that research publications should adhere to high standards of transparency and robustness in their methods and results. This, in turn, supports the principle of reproducibility, which is a foundation of good research, especially in the natural sciences.

The Methods section should contain sufficient detail such that the work could be repeated. It is preferable that all key methods be included in the main manuscript, rather than in the Supplementary Information. Please avoid use of “as described previously” or similar, and instead detail the specific methods used, with appropriate attribution.

Please note that Nature Portfolio journals allow unlimited space for Methods.

Statistical reporting

Wherever statistics have been derived (e.g. error bars, box plots, statistical significance) figure legends should provide and define the n number (i.e. the sample size used to derive statistics) as a precise value (not a range), using the wording “n=X biologically independent samples/animals/cells/independent experiments/n= X cells examined over Y independent experiments” etc. as applicable. The figure legends must also indicate the statistical test used. Where appropriate, please indicate in the figure legends whether the statistical tests were one-sided or two-sided and whether adjustments were made for multiple comparisons. For null hypothesis testing, please indicate the test statistic (e.g. F, t, r) with confidence intervals, effect sizes, degrees of freedom and P values noted.

All error bars need to be defined in the figure legends (e.g. SD, SEM) together with a measure of center (e.g. mean, median). For example, the legends should state something along the lines of “Data are presented as mean values +/- SEM” as appropriate. All box plots need to be defined in the legends in terms of minima, maxima, center, bounds of box and whiskers and percentile.

For examples of expected description of statistics in figure legends, please see the following: https://www.nature.com/articles/s41467-019-11636-5 or https://www.nature.com/articles/s41467-019-11510-4.

When describing results as "significant" in the main text, please include details about the statistical test used and provide an exact p-value, rather than a significance threshold.

Please note that statistics such as error bars significance and p values cannot be derived from n<3 and must be removed in all such cases.

We strongly discourage deriving statistics from technical replicates, unless there is a clear scientific justification for why providing this information is important. Conflating technical and biological variability, e.g., by pooling technically replicates samples across independent experiments is strongly discouraged.

For examples of expected description of statistics in figure legends, please see the following: https://www.nature.com/articles/s41467-019-11636-5 or https://www.nature.com/articles/s41467-019-11510-4.

Data presentation

When choosing a color scheme please consider how it will display in black and white (if printed), and to users with color blindness. Please consider distinguishing data series using line patterns rather than colors, or using optimized color palettes such as those found at https://www.nature.com/articles/nmeth.1618 The use of colored axes and labels should be avoided. Please avoid the use of red/green color contrasts, as these may be difficult to interpret for colorblind readers.

Bar graphs should only be used to present counts or proportions. If you are using bar graphs that present means/averages, it is best practice to include individual data points and/or convert the graph to a boxplot or dot-plot. You may wish to refer to this blog post (https://ecrlife420999811.wordpress.com/2018/07/10/beyond-bar-graphs-free-tools-and-resources-for-creating-more-transparent-figures-for-small-datasets/) about representing data distribution in plots (particularly for small datasets).
