# Supplementary material for: Comparative analysis of genome-scale, base-resolution DNA methylation profiles across 580 animal species
Source: Nat Commun. 2023 Jan 16;14:232. doi: 10.1038/s41467-022-34828-y (PMC9842680; doi:10.1038/s41467-022-34828-y)
Supplement: Supplementary file 3 — Description of Additional Supplementary Files [file 41467_2022_34828_MOESM3_ESM.pdf]

DESCRIPTION OF ADDITIONAL FILES

Comparative analysis of genome-scale, base-resolution DNA methylation profiles across 580 animal species

Supplementary Data

Supplementary Data 1. Annotation data and DNA methylation profiling statistics for all processed samples

Supplementary Data 2. Annotation data for the animal species included in this study

Supplementary Data 3. Annotation data for the individual animals included in this study

Supplementary Data 4. Annotation data and DNA sequencing statistics for the unconverted libraries
